# Toward Earth system modeling with resolved clouds and ocean submesoscales on heterogeneous many-core HPCs

**DOI:** 10.1093/nsr/nwad069

**Published:** 2023-03-20

**Authors:** Shaoqing Zhang, Shiming Xu, Haohuan Fu, Lixin Wu, Zhao Liu, Yang Gao, Chun Zhao, Wubing Wan, Lingfeng Wan, Haitian Lu, Chenling Li, Yanfei Liu, Xiaojing Lv, Jiayu Xie, Yangyang Yu, Jun Gu, Xuantong Wang, Yan Zhang, Chenhui Ning, Yunlong Fei, Xiuwen Guo, Zhaoying Wang, Xue Wang, Zhenming Wang, Binglin Qu, Mingkui Li, Haoran Zhao, Yingjing Jiang, Guang Yang, Lv Lu, Hong Wang, Hong An, Xin Zhang, Yu Zhang, Wentao Ma, Fujiang Yu, Jing Xu, Xiaopei Lin, Xueshun Shen

**Affiliations:** Key Laboratory of Physical Oceanography, Ministry of Education/Institute for Advanced Ocean Study/Frontiers Science Center for Deep Ocean Multispheres and Earth System (DOMES), College of Oceanic and Atmospheric Sciences, Ocean University of China, Qingdao 260003, China; Laoshan Laboratory, Qingdao 266201, China; Ministry of Education Key Lab for Earth System Modeling, and Department of Earth System Science, Tsinghua University, Beijing 100084, China; National Supercomputing Center in Wuxi, Wuxi 214100, China; Ministry of Education Key Lab for Earth System Modeling, and Department of Earth System Science, Tsinghua University, Beijing 100084, China; National Supercomputing Center in Wuxi, Wuxi 214100, China; Key Laboratory of Physical Oceanography, Ministry of Education/Institute for Advanced Ocean Study/Frontiers Science Center for Deep Ocean Multispheres and Earth System (DOMES), College of Oceanic and Atmospheric Sciences, Ocean University of China, Qingdao 260003, China; Laoshan Laboratory, Qingdao 266201, China; Ministry of Education Key Lab for Earth System Modeling, and Department of Earth System Science, Tsinghua University, Beijing 100084, China; National Supercomputing Center in Wuxi, Wuxi 214100, China; Frontiers Science Center for Deep Ocean Multispheres and Earth System, and Key Laboratory of Marine Environment and Ecology, Ministry of Education, Ocean University of China, Qingdao 266100, China; Laoshan Laboratory, Qingdao 266201, China; Deep Space Exploration Laboratory/School of Earth and Space Sciences, University of Science and Technology of China, Hefei 230026, China; National Supercomputing Center in Wuxi, Wuxi 214100, China; Key Laboratory of Physical Oceanography, Ministry of Education/Institute for Advanced Ocean Study/Frontiers Science Center for Deep Ocean Multispheres and Earth System (DOMES), College of Oceanic and Atmospheric Sciences, Ocean University of China, Qingdao 260003, China; Laoshan Laboratory, Qingdao 266201, China; National Supercomputing Center in Wuxi, Wuxi 214100, China; National Supercomputing Center in Wuxi, Wuxi 214100, China; National Supercomputing Center in Wuxi, Wuxi 214100, China; National Supercomputing Center in Wuxi, Wuxi 214100, China; National Supercomputing Center in Wuxi, Wuxi 214100, China; Key Laboratory of Physical Oceanography, Ministry of Education/Institute for Advanced Ocean Study/Frontiers Science Center for Deep Ocean Multispheres and Earth System (DOMES), College of Oceanic and Atmospheric Sciences, Ocean University of China, Qingdao 260003, China; Deep Space Exploration Laboratory/School of Earth and Space Sciences, University of Science and Technology of China, Hefei 230026, China; Ministry of Education Key Lab for Earth System Modeling, and Department of Earth System Science, Tsinghua University, Beijing 100084, China; Ministry of Education Key Lab for Earth System Modeling, and Department of Earth System Science, Tsinghua University, Beijing 100084, China; Ministry of Education Key Lab for Earth System Modeling, and Department of Earth System Science, Tsinghua University, Beijing 100084, China; Key Laboratory of Physical Oceanography, Ministry of Education/Institute for Advanced Ocean Study/Frontiers Science Center for Deep Ocean Multispheres and Earth System (DOMES), College of Oceanic and Atmospheric Sciences, Ocean University of China, Qingdao 260003, China; Frontiers Science Center for Deep Ocean Multispheres and Earth System, and Key Laboratory of Marine Environment and Ecology, Ministry of Education, Ocean University of China, Qingdao 266100, China; Key Laboratory of Physical Oceanography, Ministry of Education/Institute for Advanced Ocean Study/Frontiers Science Center for Deep Ocean Multispheres and Earth System (DOMES), College of Oceanic and Atmospheric Sciences, Ocean University of China, Qingdao 260003, China; Key Laboratory of Physical Oceanography, Ministry of Education/Institute for Advanced Ocean Study/Frontiers Science Center for Deep Ocean Multispheres and Earth System (DOMES), College of Oceanic and Atmospheric Sciences, Ocean University of China, Qingdao 260003, China; Key Laboratory of Physical Oceanography, Ministry of Education/Institute for Advanced Ocean Study/Frontiers Science Center for Deep Ocean Multispheres and Earth System (DOMES), College of Oceanic and Atmospheric Sciences, Ocean University of China, Qingdao 260003, China; Frontiers Science Center for Deep Ocean Multispheres and Earth System, and Key Laboratory of Marine Environment and Ecology, Ministry of Education, Ocean University of China, Qingdao 266100, China; Key Laboratory of Physical Oceanography, Ministry of Education/Institute for Advanced Ocean Study/Frontiers Science Center for Deep Ocean Multispheres and Earth System (DOMES), College of Oceanic and Atmospheric Sciences, Ocean University of China, Qingdao 260003, China; Laoshan Laboratory, Qingdao 266201, China; Key Laboratory of Physical Oceanography, Ministry of Education/Institute for Advanced Ocean Study/Frontiers Science Center for Deep Ocean Multispheres and Earth System (DOMES), College of Oceanic and Atmospheric Sciences, Ocean University of China, Qingdao 260003, China; Key Laboratory of Physical Oceanography, Ministry of Education/Institute for Advanced Ocean Study/Frontiers Science Center for Deep Ocean Multispheres and Earth System (DOMES), College of Oceanic and Atmospheric Sciences, Ocean University of China, Qingdao 260003, China; Key Laboratory of Physical Oceanography, Ministry of Education/Institute for Advanced Ocean Study/Frontiers Science Center for Deep Ocean Multispheres and Earth System (DOMES), College of Oceanic and Atmospheric Sciences, Ocean University of China, Qingdao 260003, China; Key Laboratory of Physical Oceanography, Ministry of Education/Institute for Advanced Ocean Study/Frontiers Science Center for Deep Ocean Multispheres and Earth System (DOMES), College of Oceanic and Atmospheric Sciences, Ocean University of China, Qingdao 260003, China; Key Laboratory of Physical Oceanography, Ministry of Education/Institute for Advanced Ocean Study/Frontiers Science Center for Deep Ocean Multispheres and Earth System (DOMES), College of Oceanic and Atmospheric Sciences, Ocean University of China, Qingdao 260003, China; Laoshan Laboratory, Qingdao 266201, China; School of Computer Science and Technology, University of Science and Technology of China, Hefei 230026, China; CEAKJ ADPRHexa Inc., Shaoguan 512026, China; National Marine Environmental Forecasting Center, Beijing 100081, China; State Key Laboratory of Satellite Ocean Environment Dynamics, Second Institute of Oceanography, Ministry of Natural Resources, Hangzhou 310012, China; National Marine Environmental Forecasting Center, Beijing 100081, China; Chinese Academy of Meteorological Sciences, Beijing 100081, China; Key Laboratory of Physical Oceanography, Ministry of Education/Institute for Advanced Ocean Study/Frontiers Science Center for Deep Ocean Multispheres and Earth System (DOMES), College of Oceanic and Atmospheric Sciences, Ocean University of China, Qingdao 260003, China; Laoshan Laboratory, Qingdao 266201, China; The Center of Earth System Modeling and Prediction, China Meteorological Administration, Beijing 100081, China

**Keywords:** high-resolution Earth system models, heterogeneous supercomputer, cloud and submesoscale resolving, weather-climate extremes, finer cross-scale interactions

## Abstract

With the aid of the newly developed ‘Sunway’ heterogeneous-architecture supercomputer, which has world-leading HPC (high-performance computer) capability, a series of high-resolution coupled Earth system models (SW-HRESMs) with up to 5 km of atmosphere and 3 km of ocean have been developed. These models can meet the needs of multiscale interaction studies with different computational costs. Here we describe the progress of SW-HRESMs development, with an overview of the major advancements made by the international Earth science community in HR-ESMs. We also show the preliminary results of SW-HRESMs with regard to capturing major weather-climate extremes in the atmosphere and ocean, stressing the importance of permitted clouds and ocean submesoscale eddies in modeling tropical cyclones and eddy-mean flow interactions, and paving the way for further model development to resolve finer scales with even higher resolution and more realistic physics. Finally, in addition to increasing model resolution, the development procedure for a non-hydrostatic cloud and ocean submesoscale resolved ESM is discussed, laying out the major scientific directions of such a huge modeling advancement.

## Teaser

A series of ultra-fine resolution coupled Earth system models permitting clouds and ocean submesoscales have been developed based on heterogeneous many-core HPCs. These high-resolution simulations in general exhibit substantially improved capability of delineating major weather-climate extremes in the atmosphere and ocean, paving for developing more advanced models that can foretell more detailed atmosphere and ocean activities.

## INTRODUCTION

A coupled Earth system model (ESM) simulates the interactions of atmosphere, ocean, sea-ice and land processes, and derives the variations of the Earth system through combining these component fluids together [1]. An ESM is an important tool to advance the understanding of the mechanisms of variability and change in the Earth climate system [2,3]. Combined with the Earth observing system, coupled ESMs can be used to more accurately predict future states of the Earth system [4]. Nowadays, developing high-precision coupled ESMs has become an indispensable part of Earth science advancement.

Due to the existence of multiscale processes in component fluids and interactions among them, the energies cascading (from larger scales to smaller scales) and invert cascading (from small scales to larger scales) are fundamental features in the evolution of Earth system states through interactions of scales [5]. This means that enhanced modeling of the Earth system shall resolve on scales as fine as possible, within the availability of high-performance computing (HPC) resources. Therefore, the development of high-resolution (HR) coupled ESMs is a major direction for the advancement of Earth climate sciences and for linking fundamental research and applications for societal services. An outstanding example is the U.S. Energy Exascale Earth System Model (E3SM) project (https://climatemodeling.science.energy.gov/projects/energy-exascale-earth-system-model-e3sm), which pursues a new ESM consisting of a very fine resolution and non-hydrostatic atmosphere [6] and ocean components based on new unstructured mesh systems.

The development of coupled ESMs is considered one of the greatest scientific achievements of the 20th century as it brings deep understanding of the various complex processes of the Earth climate system and realizes robust numerical weather predictions [7] and reliable long-time climate assessment (Coupled Model Intercomparison Project, CMIP 3-6) [1,2,8,9]. However, the efforts of model development are confronted with serious challenges on different fronts. On the one hand, the traditional homogeneous-architecture multi-core HPC on which ESMs used to rely has reached a physical ceiling, along with the need for an HR ESM, and a transition to new heterogeneous-architecture many-core systems is needed to continuously increase computing power with reduced unit power consumption [10]. On the other hand, many scientific issues have arisen on the development frontier [11]. The major ones include: (i) the atmosphere and ocean components in most ESMs are based on the hydrostatic balance, which has limitations with regard to accurately describing meso- and small-scale processes [12–15]; (ii) sea-ice modeling has many uncertainties, thus adversely impacting on climate assessment and future projections [16–19]; (iii) modeling of biogeochemical processes and their interactions with oceanic and atmospheric environments contains uncertainties [20–23]. Once the HPC capability allows scientists to address these challenges, HR coupled Earth system model (HR-ESM) development will be so important that as well as advancing Earth sciences and promoting societal services, it will also drive the birth of new HPC systems that have more computing power and less energy consumption.

Making full use of the newly developed ‘Sunway’ heterogeneous supercomputer in China, which has a world-leading HPC capability, the Laoshan Laboratory (LaoshanLab) is making great efforts to organize a large group of domestic scientists to overcome these challenges in the development of SW-HRESMs, based on the earlier effort of the International Laboratory for High-Resolution Earth System Prediction (iHESP). While reviewing the major advancement of the international Earth science community in these relevant fields, this study describes the process and progress of the LaoshanLab-led large group in the development of SW-HRESMs. Finally, after describing ongoing development, the study also lays out the major directions of future studies that are looking to pursue advances in fundamental Earth climate science: how do fine cross-scale interactions impact on the predictability of the Earth system?

## DEVELOPMENT OF COUPLED ESMs AT DIFFERENT GRID SPACINGs

Based on the powerful newly developed heterogenous Sunway HPC platform (see Methods), we construct the SW-HRESM framework based on CESMHR_sw1.0 and CESM2 (see Methods and Text S2). Then we establish four ocean-ice models with nominal resolutions of 0.15° (∼15 km), 0.1° (∼10 km), 0.05°(∼5 km) and 0.03° (∼3 km) on the new TS (Tripolar ocean/sea-ice grid based on Schwarz-Christoffel conformal mapping) grid system, referred to as TS015, TS010, TS005 and TS003 (see Table S1 for detailed features), respectively, with enhanced delineation of detailed features of submarine topography (Figs S2 and S3), and three atmosphere (land) models with nominal resolutions of ∼12 km, 9 km and 5 km on the cubic-sphere mesh system, referred to as ne240, ne360 and ne480, respectively. It is worth mentioning that the CESM2 land model includes biogeochemical processes such as plant nitrogen and carbon cycling as well as plant photosynthesis, which enable studies on the cycles of carbon, nitrogen, phosphorus, etc. of the land ecosystem. With these atmosphere and ocean models, we develop a series of coupled SW-HRESMs to meet the needs of multiscale studies in the weather-climate sciences as well as studies on the carbon cycling of the Earth system that have different computational costs.

### Aquaplanet experiments for the HR-atmosphere models and the AMIP configuration

We first conduct the aquaplanet experiments using the series of HR-atmosphere models to understand the fundamental behaviors of these HR-atmosphere models. Once reasonable results are gained from the aquaplanet experiments (see Text S4), based on the 1 arc-minute ($\frac{1}{{60}}$°) resolution Earth Topography and Bathymetry data set ETOPO1 [24], we set the standard Atmospheric Model Intercomparison Project (AMIP) configuration [25] for topography and land/soil type as well as radiation forcings etc. Compared to the previously developed ne120 model [10,11], the Earth surface terrains in ne240, ne360 and ne480 models are resolved more and more precisely in the terrain shapes (Fig. S5) and heights (Fig. S6). Then we establish numerical experiment cases of the atmosphere for the pre-industrial control (PIC) simulations. We first integrate the PIC atmosphere numerical experiments of ne240, ne360 and ne480 for at least 10 days to ensure the stability of each HR-atmosphere model so that all of them are ready for the coupled simulations discussed later.

### Ocean and sea-ice model simulations at different resolutions

Starting from the Polar Science Center Hydrographic Climatology (PHC) [26] and forced with the normal-year forcing from Coordinated Ocean-ice Reference Experiments (CORE) [27], and with the increase in model resolution producing more precisely resolved submarine topography features (Figs S2 and S3), the TS015, TS010, Ts005 and TS003 models are integrated for three years. The mean sea surface vorticity on the last day is shown in Fig. S8. The major vorticity distributes over the western boundary current (WBC) areas in the western Pacific and Atlantic as well as Indian Oceans. As the model resolution increases, the simulated mesoscale eddies around the Kuroshio and Kuroshio extension (KE) and Atlantic WBC areas become stronger, especially when comparing the simulation at 3 km resolution (Fig. S8d) with that at 15 km (Fig. S8a).

#### Eddy kinetic energy and mesoscale and submesoscale eddies

**Figure 1.**
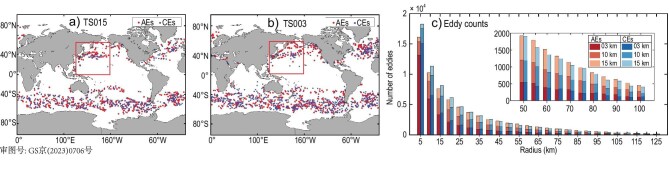
(a and b) Spatial distributions of eddy centroids for anticyclonic eddies (AEs) and cyclonic eddies (CEs) with amplitude >0.05 m, detected from (a) TS015 and (b) TS003 models. The red boxes denote the regions with SST errors in Fig 3e and f. (c) Histogram of eddy radius constructed from TS015, TS010 and TS003 for the last year of a three-year model spin-up. The radius range in 50–100 km is zoomed-in in the upper-right corner.

The mean surface eddy kinetic energy (EKE) in different resolution simulations (Fig. S9) shows that mesoscale eddies are more energetic in regions with stronger currents. Consistent with previous studies [28–30], the magnitude of EKE in WBCs is strongly dependent on model resolutions. The EKE distributions are also consistent with the distributions of major eddies (with an amplitude >0.05 m) as the model resolves finer scales with higher resolution as shown in Fig. 1, for which an automated eddy detection algorithm [31] is applied to model outputs. We see that the count of eddies with a radius of <20 km significantly increases (Fig. 1c) as the model resolves the scales that permit submesoscale activities. The distribution of mesoscale features in a model mainly depends on the ratio of the first baroclinic deformation radius (*R*_d_) to the horizontal grid spacing [32]. When the grid spacing is several times smaller than *R*_d_, barotropic and baroclinic instability processes can be properly resolved [33]. As expected, there are more eddies formed through barotropic and baroclinic instabilities as the model horizontal resolution is increased. Moreover, previous studies argue that one actually needs to significantly increase the resolution (∼0.01°) in order to resolve the submesoscale instabilities that can energize the mesoscale [34,35].

From further analyses on eddy activities (see Text S5), we may conclude that the models that permit ocean submesoscales improve the representation for multiscale ocean circulations. Elucidating the interactions between mesoscale eddies and submesoscale activities is an important step for understanding submesoscale-resolved modeling. Next, we will analyze the impact of eddy-mean flow interactions with permitted submesoscales on sea surface temperature (SST) simulation to further address this point.

#### Sea surface temperatures in submesoscale-permitted models

To understand the response of the ocean model at different resolutions to climatological atmospheric forcing, we first use the 38-year (1982–2019) 0.25^o^ resolution Advanced Very-High-Resolution Radiometer (AVHRR) SST product to establish the observed SST time mean, and use model data from the last two years to compute the model SST time ‘mean’, and then we calculate the SST ‘mean’ errors produced by different HR models. Although three-year model integrations may be too short for a direct evaluation of SST bias which requires a long-time integration as in the previous modeling studies (e.g. [11]), these results play a vital role in strengthening our understanding of the impacts of fine-scale eddy activities on SST simulation as the resolution of models increases. First we found, as model resolution increases, that the SST errors in the tropics and subtropics consistently became smaller, but that large uncertainties exist at high latitudes, especially the high-latitude Southern Ocean and North Atlantic (Fig. S12). This is consistent with the change of SST bias when the 25 km resolution model is compared with the 100 km resolution model [11]. It is interesting that the SST errors in the region from the Norwegian Sea to Barents Sea consistently decrease as the model resolution increases. From scale separation analyses (see Text S6), we understand that, in tropical and subtropical oceans as well as the region from the Norwegian Sea to Barents Sea, due to the dominant mechanism of eddy-mean flow interactions for local upper oceans [36–38], well-represented fine-scale eddies in the HR models improve the SST simulation [39,40]. In these regions, submesoscale eddies and fronts are active because of complex topography features and the existence of WBCs [38,41]. Nevertheless, for the Southern Ocean and North Atlantic, slowly varying processes such as the interactions between the upper and deep oceans [42], as well as the global thermohaline transport [43], play large roles. Although large SST simulation uncertainties still exist in these regions within interannual integrations, as such submesoscale-permitted models have long-time integrations, globally reduced SST bias can be expected because of the positive impacts of more accurate local effects of ocean mixing [44] and eddy-mean flow interactions [45] on large-scale circulations. It is noteworthy that the three-year integrations are to a certain extent too short for SST evaluation, and more comparisons are needed in future once long-time simulations (a few decades, for instance) are available at such high resolutions. See more detailed analyses in Text S6, based on Figs S12–16.

### Challenges and issues in the high-resolution coupling of atmosphere and ocean

Using the remapping function of the Earth system modeling framework (ESMF) tool, we prepare the mapping coefficient matrices of exchange fluxes between the atmosphere and ocean and then construct the coupled models. To understand the issues and detect problems during the development of HR coupled models, for a fixed-resolution component of atmosphere or ocean, we set up a series of resolutions in its counterpart of ocean or atmosphere. For example, we use the ne480 Community Atmosphere Model-Spectral Element (CAM-SE) atmosphere component to couple with ocean components at resolutions of 0.15^o^, 0.1^o^, 0.05^o^ and 0.03^o^, namely 5v15, 5v10, 5v5 and 5v3, respectively. Similarly, the ocean component of Parallel Ocean Program (POP) at a resolution of 0.05^o^ is applied to couple with atmospheric components at resolutions of 25 km, 12 km and 9 km, namely 25v5, 12v5 and 9v5. It is worth emphasizing that such a development track with a sequence of grid spacings is very important to ensure the success of an ultrafine resolution of 5v3 (ne480 CAM-SE coupled with 0.03^o^ resolution POP) HR. For example, we can compare the new 25v10 coupled model that uses the CESM2 and TS ocean and sea-ice grids with the old 25v10 CESM1.3 that has long-time stable integrations to ensure everything is comprehensible during the gridding system upgrade. We summarize the challenges and problems detected and resolved through tests and examinations of the series of HR coupled models in the following two ways.

#### Coupling technical issue: consistent mask and physical features in exchange flux remapping

As the model resolution increases, the detailed structure of coastal lines can be delineated, and so ensuring a consistent coastal line between components of atmosphere and ocean becomes very important. In some complex meandering sections, the water/land mask may not be consistent with the model physical data due to the existence of the uncertainty of floating-point if-statements. Usually, the higher the model resolution is, the more chance that the issue will occur. Under the circumstances, we first need to detect the locations and manually correct the inconsistence. Most cases can be detected by the fraction check of remapping coefficients of exchange fluxes at the model initialization stage. However, in some special circumstances, especially in the 5v3 model, as the geographical locations of certain atmosphere and ocean grid-points are very (infinitively) close, although the fraction check is passed, the inconsistency of the model mask and physical data still exists. This could cause the model to be unstable due to the accumulation of exchange flux interpolation errors. We found a few cases occurring at the coastal line in the north of Europe and south of Asia for the 5v5 and 5v3 models. It may take quite a while to locate such a problem since the cause and effect could be non-synchronous and occur at different places due to the propagation of errors. Once perfectly consistent numerical expression of complicatedly meandering coastal lines is established in HR coupled models, the correctly calculated air–sea exchange fluxes are ensured.

#### Coupling science issue: physical balance of atmosphere and ocean processes in air–sea interactions

We usually make an initial condition for a newly constructed HR coupled model (a 5v3 model in this case, for instance) by re-gridding the coupled state of an existing lower-resolution model (i.e. the old 25v10 grid in this case) that has established long-time simulations [11]. If the HR coupled model directly starts from a re-gridded lower-resolution atmosphere-ocean coupled model state, numerical instability could develop due to the physical imbalance of atmosphere and ocean processes at the air–sea interface because of the very different spin-up timescales between atmosphere and ocean. Figure S18 gives an example; as the 5v3 model is initialized from the re-gridded 646th-yr coupled state of the old 25v10 long-time PIC simulation, it becomes unstable over the Davis strait (Fig. S18a). Specifically, while local atmosphere processes are quickly developed as a response to the local warm water, ocean processes are adjusted slowly, especially when the vertical mixing is involved. Due to the blocking effects of topography at the west of the strait, the quickly developed east wind makes an accumulation of warm water at the west coastal area, which further strengthens the convection in the intersection area of red-dotted and black-dotted channels. Such a coupling feedback effect makes the model become quickly unstable and blow up. To fix this issue, the HR ocean and sea-ice components (e.g. 5v3) are designed to spin up for at least 20 days with specified surface fluxes (for example, using the fluxes on 1 January 646-yr of 25v10). After that, the model becomes stable since a sufficient adjustment has been gained in the local ocean processes.

## PRELIMINARY RESULTS FROM THE SERIES OF SW-HRESMS

We re-grid the ocean and sea-ice states on 1 January 646-yr of the 25v10 long-time PIC simulations [11], to form the ocean and sea-ice initial conditions for ocean and sea-ice coupled models at four different spatial resolutions (Table S1). Then, we integrate the ocean and sea-ice models for 20 days using the instantaneous 25v10 atmospheric fluxes on 1 January 646-yr, and then integrate the newly developed coupled ESMs 12v5, 9v5, 5v15, 5v10, 5v5 and 5v3 for 6 months. We use these six-month data to conduct preliminary evaluations for these models. Our purpose is to gain preliminary insights into the influences of model resolutions and computational costs on simulation features and establish a baseline for the selection of model resolutions dealing with different weather or scientific climate issues.

### Sea surface temperatures and extreme rainfalls

**Figure 2.**
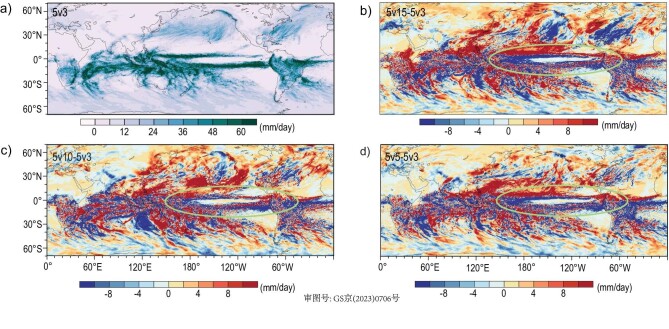
The distributions of (a) extreme rainfalls in the 5v3 model, as well as the difference in extreme rainfalls between the (b) 5v15 and 5v3 (5v15-5v3), (c) 5v10 and 5v3 (5v10-5v3) and (d) 5v5 and 5v3 (5v15-5v3) models. The extreme rainfall at each grid point is defined as the average top 10% daily precipitation amount during the last 90 days of the half-year model integrations.

The SST links the atmosphere, ocean and sea ice together and exerts an impact on land processes through an influence on atmosphere, and it is therefore a very important physical field in the Earth system. Through the enhancement of spatial resolutions in the ocean component, more meso- and small-scale activities appeared in the WBC areas of the northwest Pacific and Atlantic as well as the Antarctic Circumpolar Current areas (Fig. S19), where the same resolution atmosphere model (CAM-SE ne480) was coupled with four different resolutions in the ocean components to form 5v15, 5v10, 5v5 and 5v3 coupled models. The other distinctive feature is that a more detailed structure of tropical instability waves (TIWs) at equatorial areas is observed along with an increase in ocean resolution. Defining the extreme rainfall at each grid point as the average amount of the top 10% of daily precipitation during the last 90 days of simulations, we show the distributions of such extreme rainfalls in Fig. 2. Consistent with the SST features, even though we used exactly the same atmosphere model, the extreme rainfalls appear more locally and stronger, especially over equatorial areas (green ellipses).

The atmospheric resolution of 5 km is close to convection permitting, which not only improves the intensity and spatial distribution of extreme precipitation but can also reduce the source of uncertainty [46,47]. In addition, for areas rich in precipitation, such as South America, especially the abundant Amazon rain forest, a previous study [11] found that mean precipitation tends to be underestimated in both ne120 (25v10) and ne30 (∼1° by 1° for atmosphere and ocean), but the negative bias in the higher resolution simulations (e.g. 25v10) becomes weaker. A consistent phenomenon is discernable based on even higher resolution simulations (Fig. 2). Specifically, the intensities of both mean and extreme precipitation gradually strengthen along with an increase of ocean resolution (5v15, 5v10, 5v5 and 5v3), yielding the strongest precipitation intensity in 5v3 simulations and weakest in 5v15 simulations. The enhanced precipitation along with the improved representation, such as submesoscale ocean eddies, is possibly linked to the teleconnection effect between SST in the Atlantic and Pacific oceans and precipitation in South America [48,49]. Rainfall in the Amazon region is crucial to the health of the rainforest as the ‘lungs of the Earth’ [50]. Considering a general negative bias of precipitation in climate models in this region [51], the potentially reduced negative precipitation bias through the increase of ocean spatial resolution pinpoints the importance of HR-ESMs in understanding the climate effect on extreme precipitation over this area.

### Atmospheric circulations and tropical cyclones

**Figure 3.**
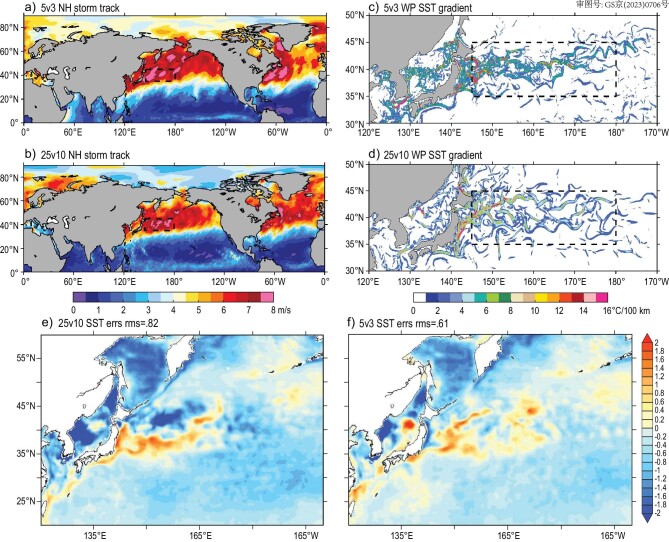
The distributions of (a and b) atmosphere storm track indices defined by the standard deviation of surface v-velocity 24-hour variations. (c and d) Corresponding SST gradient norms, produced by 5v3 (a, c and f) and 25v10 (b, d and e) models, in the last three months of the half year of spin-up integrations. (e and f) Half-year SST mean errors in the northwest Pacific Ocean in the 25v10 (panel e) and 5v3 (panel f) models.

We use the features of atmosphere storm tracks (ASTs) to show the behavior of atmospheric circulations simulated at different resolutions. ASTs are defined as the regions where synoptic eddy activities are strongest [52], being closely related to extratropical-cyclone and anticyclone activities that affect local intense wind or precipitation [53,54]. The Northern Hemisphere ASTs exhibit distinct seasonal variations, with major interannual variability in boreal winter significantly influencing most of the inhabited environments on Earth [55,56]. The ASTs are the product of the responses of atmosphere dynamics to underlying surface conditions [57]. Under better representation of meso- and small-scale SST structures in the ocean models, HR models improve the simulation of ASTs compared to the low-resolution case [58], in which the resolutions of both the atmosphere and ocean components are ∼100 km. As shown in Fig. 3, the simulated results from 5v3 show more centralized and stronger AST structures compared to those in 25v10 simulations (compare panel a to panel b), as well as stronger and more concentrated SST gradient norms around the WBCs in the Kuroshio and Kuroshio extension areas of the West Pacific (panels c and d), which link to stronger ASTs over there [59]. It is worth noticing that due to enhanced representation for fine-scale eddy activities, the 5v3 model has significantly reduced SST errors in this region (see Fig. 3e and f).

**Figure 4.**
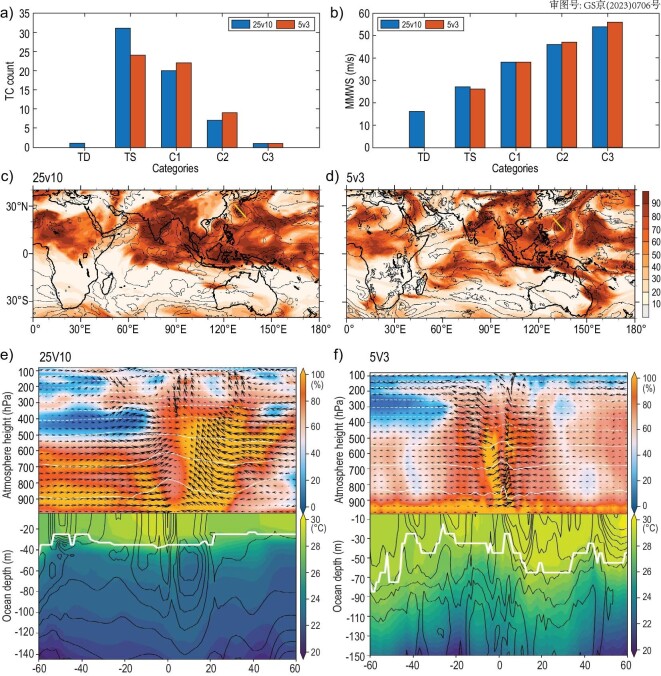
(a and b) Distributions of (a) TC counts and (b) mean maximum wind speed (MMWS) in different categories produced by the 5v3 model in January–October as the model is initialized from the 25v10 state on 1 January 0646-year, compared with the results of the 25v10 model in the same period. (c and d) Distributions of 850 hPa relative humidity (shaded) and wind speed (contours) in the 25v10 model (c) at 00UTC of 2 September, and 5v3 model at 06UTC of 31 August, as the C3 category TC (marked by the yellow segment in each panel) in both models reaches its maximum wind speed. (e and f) The atmosphere and ocean conditions at the air–sea interface in the vertical section of the TC marked by the thick yellow segment in panels c (for 25v10) and d (for 5v3). The atmosphere (ocean) relative humidity (%) (temperature: ^o^C) is color shaded, while the atmosphere (ocean) temperature (unit: ^o^C) (salinity: psu) is contoured, and the vector arrows always represent the atmospheric velocities (u, w × 10 ^2^) (unit: m/s) (ocean currents: 0.04 m/s). The white-bold line represents the mixing layer depth.

For detecting the behavior of the new HR coupled models on tropical cyclones (TCs), we extend the 5v3 model to the end of October from the half-year spin-up run to cover major Northern Hemisphere TC months [60]. We show the 5v3 model TC statistics in these 10 months in Fig. 4a and b with the 646-year 25v10 results in the same period as a reference. Usually, a low-resolution (e.g. ∼100 km) model is incapable of representing TC, and usually underestimates TC count and intensity significantly [61]. When the resolution is increased to 25 km, which is equivalent to TC-resolving, a significantly improved performance of TC activities has been achieved by enhancing both the TC count and intensity, but the model still tends to overestimate TC count and underestimate TC intensity, especially in high categories [62,63], which is primarily attributable to insufficiently resolved cloud structures and their interactions with the environment [11,64,65]. Although the comparison here has no quantitative meaning, we can still see that the higher-resolution model tends to enhance the TC intensity in higher categories and reduce TC counts in lower categories.

We choose the C3 (strong) typhoon in the West Pacific simulated in both 25v10 and 5v3 models to see how spatial resolutions in atmosphere-ocean coupled models may affect the air–sea interaction. Figure 4c–f displays the atmosphere and ocean conditions on the air–sea interface when the TC passes, as well as the distributions of 850 hPa relative humidity and wind speed in the tropical area. Although the locations and intensity of the TC in the 25v10 (panel c) and 5v3 (panel d) models are not identical, we can still use them to look at the difference in the resolved coherent vertical structure of atmosphere and ocean conditions when a TC passes. We can see that as cloud is permitted [66], the 5v3 model shows more asymmetric spiral cloud structure than the 25v10 model (panel f vs. e). The TC simulated by the 5v3 model has a more distinctive eye-wall structure and the corresponding upper ocean also shows more detailed structures. It is very interesting to further examine the performance of such a cloud and ocean mesoscale-permitted model when it comes to TC genesis predictability [60], with the aid of HR coupled model initialization. These preliminary analyses assert that further studies on cloud-resolving coupled ESMs are valuable to understand the impact of multiscale interactions on the seamless weather-climate variability.

### The polar regions and sea ice

**Figure 5.**
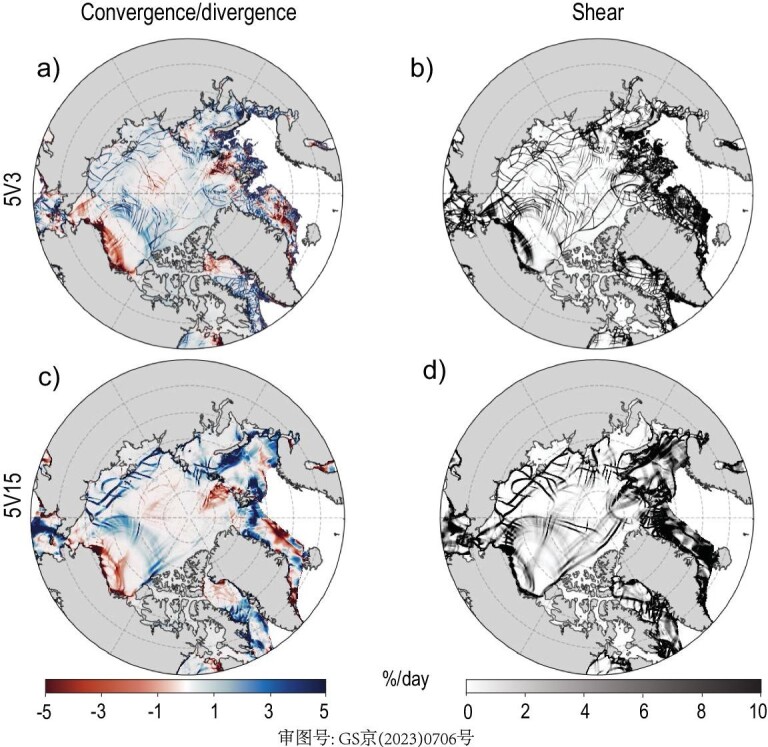
The distributions of sea-ice (a and c) convergence and (b and d) shear rates in the 5v3 (a and b) and 5v15 (c and d) models. The sea-ice linear kinematic features (LKFs) are the results after a 5-day spin-up when the identical atmosphere is still in its valid predictability as coupled with different-resolution ocean components. The sea-ice convergence and shear rates are defined as (*u*_x_ + *v*_y_) and $( {\sqrt {{{( {{u}_x - {\nu }_y} )}}^2 + {{( {{u}_y + {\nu }_x} )}}^2} } )$ respectively, and for visualization convenience, the original dimension (s^−1^) is converted to day^−1^ × 100 (%/day).

Due to the existence of complex topography structures and sea-ice modeling uncertainties, the high-precision simulation of sea-ice and polar-region climate systems is still very challenging in ESMs [67]. Given the important roles of leads and polynyas in the polar-region climate system [68,69], HR sea-ice simulation is a critical step towards constraining the uncertainties of Earth climate system modeling. Figure 5 gives a comparison of the rates of sea-ice convergence (*u*_x_ + *v*_y_) and shear $[ {\sqrt {{{( {{u}_x - {\nu }_y} )}}^2 + {{( {{u}_y + {\nu }_x} )}}^2} } ]$ produced by the 5v3 (panels a and b) and 5v15 (panels c and d) models (where u and v are the zonal and meridional velocity components of ice motion and the subscript represents the corresponding first-order derivative). There is a consistency between the large-scale sea-ice drift and deformation fields as simulated by the 0.15°, low resolution and its HR counterpart at 0.03°. The overall features as shown in Fig. 5 include the shearing across the western Arctic Basin, as well as the Beaufort Gyre circulation and the convergence and divergence associated with it. Furthermore, both models simulate multi-fractal sea-ice deformation properties, which are unique to polar oceans and ice kinematics. At such a resolution, TS003 simulates much more refined linear kinematic features (LKFs) of sea ice than TS015.

The lead networks in the Beaufort Sea and Davis Strait, as well as the shearing structure across the basin, are much narrower and more localized with TS003. Consistently, both models simulate pronounced sea-ice deformation on the ice edge, which is typical of the ice conditions and the more extensive interaction with the atmosphere and ocean. This result indicates that kilometer-scale models are capable of accurately resolving the detailed structures of sea-ice kinematics, which are key to the air–sea interaction at polar regions. Potential impacts on the larger-scale climate, and predictability, are a key research issue for future research using the new model system.

## NEW DEVELOPMENT OF NON-HYDROSTATIC AND CLOUD-RESOLVING ESM

### The Sunway 3-km-resolution iAMAS model based on MPAS-atmosphere dynamic core

**Figure 6.**
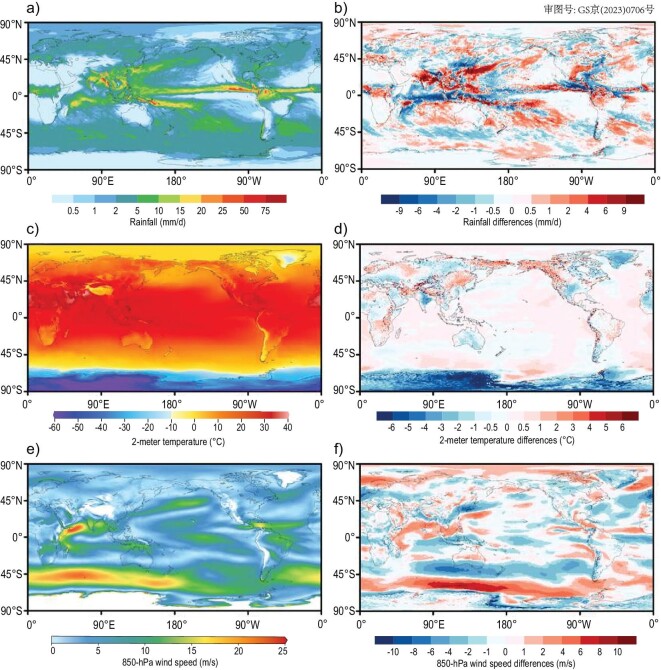
The global spatial distributions of (a) rainfall, (c) 2-meter temperature and (e) 850-hPa wind speed averaged from 12 June–10 July 2020, produced by the iAMAS with global uniform 3-km resolution starting from the ERA5 reanalysis fields at UTC 00 : 00 on 10 June 2020. For comparison, we also conduct a global uniform 60-km resolution experiment and show the corresponding differences between 3-km and 60-km resolution experiments in panels (b), (d) and (f), where both 3-km and 60-km resolution data are interpolated to 0.25^o^ × 0.25^o^ grids.

The iAMAS (integrated Atmospheric Model Across Scales) [70] is a non-hydrostatic atmospheric model that has been developed on the new Sunway heterogeneous-architecture HPC system based on the MPAS-atmosphere dynamic core, which uses a C-grid staggered unstructured Voronoi mesh system with finite-volume formation [71]. The development of iAMAS includes coding optimization such as multi-dimension-parallelism structuring, aggressive and finer-grained optimization, manual vectorization, and parallelized I/O fragmentation. It also includes restructuring model’s physical parameterizations compatible with the heterogeneous-architecture HPC environment, and implementation of an atmospheric chemistry suite [70]. Through these great efforts, the iAMAS model has been established as a non-hydrostatic, computational, high-efficiency global uniform 3-km (U3km) resolution atmosphere model on the Chinese-grown Sunway HPC platform. Compared to the 60-km (U60km) resolution version, the U3km resolution iAMAS has better representation for atmospheric meso- and small-scale dynamics and physics, characterized by stronger local rainfall, detailed topography and land-sea effects, and distinctive Antarctic Circumpolar jet-core (Fig. 6). This development paves the way for a new atmosphere component to start next-generation ESM development. It is worth mentioning that the largest difference between the U3km and U60km resolution simulations, when it comes to atmosphere temperatures and winds, is in the Antarctic area (Fig. 6d and f) because of a stronger polar vortex over the Antarctic in the U3km simulation (Fig. S20). Whether or not the strong polar vortex of such a fine-scale simulation can relax the large SST warm bias of the high-latitude Southern Ocean in coupled ESMs [72] is an interesting research topic which deserves further investigation in the future.

### The non-hydrostatic and cloud-resolving 3v3 ESM

To achieve a new-generation coupled ESM with non-hydrostatic, cloud-resolving [73] and ocean-submesoscale-eddies-permitting features, we are working on replacing hydrostatic CAM-SE with non-hydrostatic iAMAS to establish the 3v3 (3-km-resolution non-hydrostatic atmosphere coupled with the 0.03^o^ resolution POP) ESM. As a starting point, the coupling of a global 60-km-resolution iAMAS and the TS100 (1^o^ × 1^o^ resolution) POP (called 60v100) is a feasible way to establish the coupled model test platform. Then, we gradually increase the resolutions of iAMAS and POP to 15v10 and 9v3 to gain the knowledge with finer grid spacings in the coupled models, eventually focusing on the 3v3 model evaluation and optimization tuning. Once the 3v3 non-hydrostatic ESM is available, combined with the original hydrostatic version (i.e. 5v3), the impact of non-hydrostatic atmospheric simulation on synoptic scales, extended-range scales and climate scales can be more thoroughly investigated.

## SUMMARY AND DISCUSSIONS

Relying on the powerful HPC capability of the newly developed heterogeneous many-core Sunway system, a series of ESMs consisting of a variety of resolutions ∼25-, 12-, 9- and 5-km-resolution atmosphere and 15-, 10-, 5- and 3-km-resolution ocean models, namely 25v10, 12v5, 9v5, 5v15, 5v10, 5v5 and 5v3, briefly referred to as SW-HRESMs, have been developed. Being convenient and sustainable for HR coupled model development, this series of different-resolution coupled ESMs can meet the needs of research into different-scale atmosphere and ocean processes with the availability of computational resources. The evaluation of preliminary simulation results shows that enhancing the model resolution and resolving more detailed dynamical and physical processes are critical to advancing the Earth climate system modeling that drives Earth science progress and promoting a level of societal service such as early warnings for extreme weather and climate events.

Our goal is to establish a numerical study platform for the multiscale Earth system with different costs of computational resources available (Text S7). At present, both the atmosphere and ocean components in the 5v3 model assume hydrostatic balance, which has a limited capability in simulating fluid motions with fine scales in the atmosphere [15,71], especially in the planetary boundary layer, and ocean regions with complex topographical features [12]. Usually, a numerical model cannot permit any characteristics with a length scale less than two times the grid-size, while resolving a length scale requires at least four times grid-size [66]. The current atmosphere and ocean components in our 5v3 model have horizontal resolutions up to 5 km and 3 km, respectively. Such resolutions only permit the occurrence of cumulonimbus cloud cells in the atmosphere and submesoscale activities in the ocean, for which the characteristic horizontal scales are roughly 10 km and a few kilometers respectively [74,75], but do not explicitly resolve them. Seamless weather-climate studies require an ESM to represent multiscale interactions and, in particular, address the energy cascade and inverse cascade associated with finer scales.

Currently, we have the following three projects in progress.

### ESM development

The 3-km-resolution iAMAS is being applied to replace the 5-km-resolution CAM-SE in the 5v3 model to complete the development of a 3v3 coupled ESM with cloud-resolving and non-hydrostatic atmospheric simulations [73]. A new Chinese-grown basic coupler generator (BCGen) which has more user-friendly coupling interfaces, is being merged with the 5v3 model and 3v3 development. With the aid of the General Model and Data Assimilation Development Platform (G_MDA_DP) described in the following section 3, we plan to incorporate atmosphere and ocean models, including the Chinese-grown non-hydrostatic and unstructured Global-to-Regional Integrated forecast SysTem (GRIST) atmosphere model [76] and the Mass Conservation Ocean Model (MaCOM) (http://macom.oceanguide.org.cn), into the ESM. At this phase, to increase the representation of a coupled model on multiscale interactions, the parameterization of tropical sub-diurnal-scale air–sea interactions shall be addressed. Meanwhile, the parameter-optimized CoSiNE (Carbon-Silicate-Nitrogen Ecosystem) [20,77,78], an uncertainty-reduced ecosystem model that reproduces the global/regional seasonal changes of phytoplankton, revealing the mechanism of different algae affected by nutrients and zooplankton [21,22], is being incorporated into the ocean of new ESMs. Both the atmosphere and ocean in the new ESMs include biogeochemical processes that are comprehensive for studies on ecology and ca rbon cycling as well as their climate impacts. Considering the need for the biogeochemical cycle due to influences of atmospheric deposition, as well as the importance of the effect of aerosol–cloud interactions on precipitation, and of air quality on human health, the atmospheric chemistry is being implemented and tested under a 25v10 framework with different levels of complexity and will eventually be incorporated into 3v3 km ESMs. The delay in the inclusion of atmospheric chemistry is partly attributable to the fact that it is much more computationally intensive, i.e. a few times slower with the same computational resources, relative to the atmospheric physics. With the high-precision description of Earth physical environmental fields and biogeochemical processes as well as their interactions, the new ESM is expected to be a sound platform to study the evolutions of global and regional ecosystem environments as well as carbon cycle changes [79]. Then, combined with the coupled data assimilation (CDA) initialization described below, taking advantage of the grid-varying features of both atmosphere and ocean models, a seamless weather-climate prediction system with sub-kilometer scales resolved in the Asia-Pacific area is applicable.

### CDA development

The new high-efficiency CDA algorithm [80] was combined with the CESM-CDA system [81] to create an HR-ESM (25v10) CDA system. Once the 25v10 CDA reanalysis data with TCs and ocean mesoscale eddies resolved are available, we will be able to do in-depth analysis with regard to understanding the importance of incorporating various observational information from different coupled components into the HR Earth system. Then we can re-grid the 25v10 CDA results to initialize the new multi-grid unstructured ESM to carry out seamless weather-climate predictions and conduct evaluations as an initiative for seamless weather-climate predictability studies.

### Model and Data Assimilation Development Platform

With the aid of an ESMF (https://www.ncl.ucar.edu/Applications/ESMF.shtml) and the framework of BCGen, as well as the strong software management function of Git (https://github.com), implemented by the powerful C++ template of application interfaces (APIs), we are constructing a platform named G_MDA_DP. Based on the common logistics of geofluid modeling and data assimilation, the commonly structured C++ template APIs easily combine different models and data assimilation algorithms together, which substantially simplifies the engineering challenges for scientists who can then focus on the science. The G_MDA_DP shall be an excellent platform that makes the scientific development highly efficient, which will progressively advance Earth system modeling and predictions.

With the G_MDA_DP, future studies shall address the following five major challenges:

1. Higher and higher resolution Earth system modeling demands more accurate, finer cross-scale interactions in each fluid and among fluids.
2. Higher and higher resolution Earth system modeling requires more accurate description of complex underlying surface conditions and more representative planetary boundary layer processes.
3. Higher-precision ESMs require more advanced ecosystem models that have minimized uncertainties on biogeochemical processes and their feedbacks that require more precise vertical mass transport.
4. Higher and higher resolution ESMs demand more advanced CDA algorithms to create more coherent and balanced reanalysis and prediction initialization with multiscale activities.
5. Higher and higher resolution simulations and predictions demand denser and more frequently observed data for high-precision initialization and prediction verification.

## METHODS

### The new Chinese homegrown heterogeneous many-core HPC capability

Based on the architecture of Sunway TaihuLight (see Text S1), the new Sunway system is built using an upgraded heterogeneous many-core processor, SW26010P, which is similar to SW26010 in terms of architecture but has more computing cores and higher overall HPC capability. With the help of the powerful HPC capability provided by the new Sunway system, we develop a sequence of HR coupled ESMs in which the highest resolution for both the atmosphere and ocean reaches the kilometer level, toward establishing the brand-new ESM characterized by cloud resolving and the permitting of ocean submesoscales.

### The CESM2-based high-resolution Earth system modeling framework

Previously, we developed the 25 (10) km atmosphere (ocean) HR-ESM (referred to as CESM-HR_sw1.0) based on CESM1.3 on the Sunway TaihuLight HPC system with updates of some dynamic-core and parallelism modules [10,11], which exhibits substantially improved capability in reproducing climate extremes such as marine heatwaves [82], the Atlantic overturning circulation [83] and middle-high latitude air–sea interactions [84]. Making full use of such experiences and the substantially powerful capability of the new Sunway HPC system, we develop the kilometer-level HR coupled model based on CESM version 2 (CESM2) (see Text S2). Based on the CESM2 framework and powerful new Chinese homegrown heterogenous HPC platform, we first update the POP grid system to the new TS system [85], which has a smooth ramping structure for the tripolar design for higher resolutions, and we configure the ocean and sea-ice models to four different resolutions as high as nominal 0.15^o^ (TS015), 0.1^o^ (TS010), 0.05^o^ (TS005) and 0.03^o^ (TS003), covering a wide range of climate modeling and submesoscale-oriented studies (see Table S1). In Table S1, the horizontal grid scale [s = (d*x*^2^ + d*y*^2^)^1/2^] is a measure of the capability of resolving submesoscale activities. From the ocean modeling perspective, the Rossby deformation radius (R) is used as a proxy for the mesoscale [86] and investigates the capabilities of each grid. Specifically, the criterion in Hallberg (2013) [32] is adopted to justify the capability of mesoscale-resolving attained when s is smaller than half of the local value of R. The average grid scale in TS003 is roughly 1 km in the Arctic oceanic regions (north of 65^o^N) where the submesoscales are nearly resolved, and the scale of TS005 is ∼3 km, which permits the submesoscale activities to occur. Then we follow the cubic sphere CAM-SE mesh system [87] to develop a series of HR atmosphere models (new ne240, ne360 and ne480) up to a kilometer-level grid-spacing model (see Text S3). More detailed model properties are listed in Table S2. For example, a model denoted as ne480 discretizes the global atmosphere into 12441600 grid boxes with a resolution of up to ∼5 km.

## Supplementary Material

nwad069_Supplemental_Files
