# Supplementary material for: Toward Earth system modeling with resolved clouds and ocean submesoscales on heterogeneous many-core HPCs
Source: Natl Sci Rev. 2023 Mar 20;10(6):nwad069. doi: 10.1093/nsr/nwad069 (PMC10171631; doi:10.1093/nsr/nwad069)
Supplement: nwad069_Supplemental_Files [file nwad069_supplemental_files.zip › NSR_2022-1185_SW-HRESMs_Supplementary_TowardCloudResolvingESM.pdf]

Supplementary materials for

Toward Earth System Modeling with Resolved Clouds and Ocean Submesoscales on Heterogeneous Many-Core HPCs

by

Shaoqing Zhang[*1, 2], Shiming Xu[*3,4], Haohuan Fu[*3,4], Lixin Wu[*1, 2], Zhao Liu[*3,4], Yang Gao[*5,2], Chun Zhao[6], Wubing Wan[4], Lingfeng Wan[1,2], Haitian Lu[4], Chenling Li[4], Yanfei Liu[4], Xiaojing Lv[4], Jiayu Xie[4], Yangyang Yu[1], Jun Gu[6], Xuantong Wang[3], Yan Zhang[3], Chenhui Ning[3], Yunlong Fei[1], Xiuwen Guo[5], Zhaoying Wang[1], Xue Wang[1], Zhenming Wang[1], Binglin Qu[5], Mingkui Li[1,2], Haoran Zhao[1], Yingjing Jiang[1], Guang Yang[1], Lv Lu[1], Hong Wang[1,2], Hong An[7], Xin Zhang[8], Yu Zhang[9], Wentao Ma[10], Fujiang Yu[9], Jing Xu[11], Xiaopei Lin[1,2], and Xueshun Shen[12],

[1]Key Laboratory of Physical Oceanography, Ministry of Education/Institute for Advanced Ocean Study/Frontiers Science Center for Deep Ocean Multispheres and Earth System (DOMES), College of Oceanic and Atmospheric Sciences, Ocean University of China, Qingdao, 260003, China

[2]Laoshan Laboratory, Qingdao, 266201, China

[3]Ministry of Education Key Lab. for Earth System Modeling, and Department of Earth System Science, Tsinghua University, Beijing, 100084, China

[4]National Supercomputing Center in Wuxi, Wuxi, 21400, China

[5]Frontiers Science Center for Deep Ocean Multi-spheres and Earth System (FDOMES) and Key Laboratory of Marine Environment and Ecology, Ministry of Education, Ocean University of China, Qingdao, 266100, China

[6]Deep Space Exploration Laboratory/School of Earth and Space Sciences, University of Science and Technology of China, Hefei 230026, China

[7]School of Computer Science and Technology, University of Science and Technology of China, Hefei, 230026, China

[8]CEAKJ ADPRHexa Inc, Guangdong Shaoguan 512026, China

[9]National Marine Environmental Forecasting Center, Beijing 100081, China

[10]State Key Laboratory of Satellite Ocean Environment Dynamics, Second Institute of Oceanography, Ministry of Natural Resources, Hangzhou, 310012, China

[11]Chinese Academy of Meteorological Sciences, Beijing, 100081, China

[12]The Center of Earth System Modeling and Prediction, CMA, Beijing, 100081, China

Contents of this file

Text S1-S7

References S1

Figures S1-S20

Tables S1-S3

Animation S1

Text S1-S7:

Text S1: The Sunway TaihuLight heterogeneous architecture HPC system

The Chinese heterogeneous many-core HPC capability develops at a quick pace in recent decades. The Sunway TaihuLight [1, 2] is the first system to reach a peak performance of 100 PFlops in the world and the first supercomputer equipped with over 10 million computing cores. The Sunway TaihuLight was the world's fastest supercomputer for two years, from June 2016 to June 2018 according to the TOP500 lists. It was built with Chinese heterogeneous many-core processors SW26010. Each SW26010 includes 4 Management Processing Elements (MPEs), 256 Computing Processing Elements (CPEs), with the 64-bit instruction set and basic compiler components including C/C++, and FORTRAN compilers [1]. Unlike the GPU-accelerated HPC systems where data transfer has to be performed among different processors and accelerators, the on-chip heterogeneity of the SW26010 processor enables a uniform memory space to facilitate the data transfer and leads to the uniform programming model between MPE and CPEs. ESMs are generally computed in a hybrid mode to use instruction sets separately where the MPE major task (in FORTRAN) manages CPE sub-tasks (in C-language).

The new Sunway system is built using an upgraded heterogeneous many-core processor SW26010P, which is similar to SW26010 in terms of architecture but with much more total HPC capability. Taking advantage of the super powerful HPC capability of the new Sunway system, we develop a sequence of high-resolution coupled ESMs in which the highest resolution for both the atmosphere and ocean reaches the kilometer level, toward establishing the brand new ESM characterized by both the cloud resolving and ocean submesoscale eddies permitting.

Text S2: The CESM2 framework adjustable for higher resolution modeling

a, The CESM2 framework

While the detailed description of CESM2 can be found in Danabasoglu et al. (2020) [3], some fundamental features related to this study are briefly discussed here. Compared to the previous version (CESM1.3) that was used to develop the coupled 25v10 ESM [4, 5], which resolves tropical cyclones and oceanic mesoscale eddies, there are many updates for atmospheric and oceanic physics. The Community Atmosphere Model (CAM) component is updated to the version 6 (CAM6) from CAM5. The primary change includes application of the

unified turbulence scheme named Cloud Layers Unified By Binormals (CLUBB) [6, 7], updated microphysics scheme [8], new parameterization for subgrid orographic drag [9], and 70 vertical levels with the atmosphere top up to 4.5 $\times$ 10^{-6} hPa (about 130 km) etc. The ocean component is the Parallel Ocean Program Version 2 (POP2), the same as CESM1.3 but apparent physical and numerical improvements are achieved. For instance, a new parameterization is applied to the mixing effects in estuaries [10], a new parameterization for Langmuir mixing together with the component of new wave model [11]. For the sea ice component, CICE Version 5.1.2 (CICE5) [12] is applied by increasing the vertical resolution of sea-ice to eight layers from four layers in CESM1.3, which enhances the capability to resolve the profile of salinity and temperature.

The land component is updated to the version 5 of Community Land Model (CLM5) [13], which incorporates an improved representation of hydrology and ecology as well as their climate impacts through enhancing anthropogenic land use activities, as well as the addition of land-ice modeling [14, 15]. Compared to previous versions (e.g., CLM4 and CLM4.5), substantial improvements in delineating plant nitrogen dynamics have been achieved in CLM5 [3, 13, 16]. The improvements are primarily due to a few scientific advances. Firstly, the implementation of a new plant model that improves the representation of leaf and root physiology, primarily through the incorporation of plant nitrogen cycle mechanisms and nitrogen uptake traits, showing an enhanced capability in reproducing Gross primary production, leaf area index and biomass [17]. Secondly, different from traditional parameterizations for the plant photosynthetic capacity, the incorporation of a mechanistic model named Leaf Utilization of Nitrogen for Assimilation (LUNA) can explicitly calculates the response of photosynthetic capacity to environmental conditions, being able to more reasonably capture the plant photosynthetic capacity [18]. Lastly, terrestrial biosphere models alone do not in general estimate the carbon cost during plant nutrient acquisition, and the newly implemented model of the Fixation and Uptake of Nitrogen (FUN) accounts for carbon cost as a function of mycorrhizal type during N acquisition, yielding improvements of prediction in both nitrogen uptake and carbon sink [19, 20]. Overall, carbon uptake in CLM5 responds stronger to greenhouse gas such as CO2 but weaker to N fertilization, which is more realistic in comparison to observations [16, 21], enhancing the model in simulating the global carbon cycle [13].

In addition, the CESM2 includes a new tool of the Common Infrastructure for Modeling the Earth (CIME) in its coupler for communicating state information and fluxes between the model components which provides new support for land-ice coupling and some data overwriting functions [3].

***b*, A new TS grid system for kilometer-level high-resolution ocean and sea-ice models**

In this study, we use the new TS (Tripolar ocean/sea-ice grid based on Schwarz-Christoffel conformal

mapping) grid system designed by Xu et al. (2021) [22] for development of the kilometer-level resolution ocean and sea-ice model components. The new gridding system has a much better behavior in the tripolar grid configuration as shown **Fig. S1**. Unlike most of the traditional tripolar gridding systems such as TX0.1V2 [23, 24] in which the Height of T-cell East (HTE) has an unsmooth distribution (**Fig. S1a**) at the transition belt when the "longitude-latitude" lines converge to the tripolar points at the northern patch (NP), the new TS grid system applies more stereographic projection of NP and thus has much smoother transition HTEs (**Fig. S1b**) with better scalability and expandability. The TS grid system has been incorporated by Xu et al. (2021) [22] into the components of CICE and POP in the new CESM framework, establishing a good basis for our study. We test four tripolar grids of TS015, TS010, TS005 and TS003 with resolutions of nominal 0.15° (~15 km), 0.1° (~10 km), 0.05° (~5 km) and 0.03° (~3 km) with increased detailed features of submarine topography (**Figs. S2-3**), respectively, covering a wide range of climate modeling and submesoscale-oriented studies (see **Table S1**).

Text S3: The cubic sphere CAM-SE for kilometer-level atmosphere (land) model development

A local method like the spectral element (SE) method has the local domain decomposition property of the finite element (FE) method combined with high-order accuracy and the weak numerical dispersion and low numerical dissipation of spectral methods [25]. The SE method offers excellent parallel efficiency and has become the method of choice for many practical applications. The nearly-perfect scalability of SE numerical method [26] makes it plausible to use the cubic sphere CAM-SE framework [27, 28] to develop the kilometer-level high-resolution atmosphere model which includes CLM5 as its bottom boundary conditions. The cubic sphere CAM-SE framework is illustrated in **Fig. S4**.

Within the cubic sphere CAM-SE framework, the dynamical and thermodynamical equations are discretized on a cubed-sphere mesh system of the Earth surface [29] (**Fig. S4a**), for which each of 6 tiles is divided into n_e^2 elements with identical side lengths (**Fig. S4b**), where n_e represents the number of elements at each side. For example, an atmospheric model denoted by ne120np4 (n_e=120) discretizes the Earth surface as 777660 grid-boxes by 777662 grid-points, np4 represents the number of Gauss-Lobatto-Legendre (GLL) quadrature points in the interior region of each element [also meaning that the fixed degrees as 4, k and N of Lagrange-Legendre polynomials in the discontinuous Galerkin (DG) method] (see **Fig. S4c**)]. More model configurations and corresponding properties are listed in **Table S2**. For example, a model denoted as ne480np4 discretizes the global atmosphere into 12441600 grid-boxes with the resolution up to approximately 5 kilometers.

Text S4: The aquaplanet experiments for the HR-atmosphere models

The aquaplanet experiment is an excellent tool to understand the effects of modeled deep convections on the large-scale circulations [30] and multiscale variability [31]. Based on differential rotation (spherical geometry) but retaining uniform thermal forcing, the idealized model configuration can reveal the control of equilibrium deep convections, for which the tropical cyclone (TC) size in large-domain simulations of rotating radiative-convective equilibrium is a typical representative [32], including a poleward shift in TC genesis in response to warming, and the temperature dependence of TC genesis in the absence of mean circulation changes. It is a good way to look at the results of aquaplanet experiments first when we develop high-resolution atmosphere models. Beginning with the ne120 (np4 is dropped for abbreviation as a common character for all resolution settings) we developed before [4], we set three configurations with even higher resolutions of ne240, ne360 and ne480 to meet the needs of multiscale weather-climate studies under different computational costs.

Based on the standard setting of aquaplanet experiment with a uniform sea surface thermal forcing of 272.15 K [32], starting from the initial conditions of static atmospheric climatological temperature and moisture profiles, we integrate the simulations at four resolutions of ne120, ne240, ne360 and ne480 for one month. Based on the Zhang-McFarlane (ZM) convection parameterization scheme [33], we use these experiments to simply detect the features of precipitation distributions resulting from different model resolution settings, which integrate the models with static temperature and moisture profiles responding to a uniform thermal forcing. As pointed out by Merlis and Held (2019) [32], given the uniform SST thermal forcing, the model usually takes 10 days to sufficiently spinup vortices. We show the 10th-day daily accumulated rainfall of 4 different resolution models as the fundamental convection characteristics in **Fig. S7.** We see that without topography effects, the global distribution of precipitation exhibits basically symmetric characteristics in the northern and southern hemispheres around the equator. As the model resolution increases, the rainfall belts become narrower and show more detailed structure characteristics, and the symmetric double intertropical convergence zones (ITCZs) around the equator where the Coriolis parameter f is zero become more distinctive (**Fig. S7b-d** vs. **Fig. S7a**). Specifically, the simulations of ne480 show more spiral cloud structures probably due to its nature of cloud permitting.

Text S5: More analyses on ocean mesoscale eddies in different resolution models

The mean surface EKE in different resolution simulations (**Fig. S9**) shows that mesoscale eddies are more

energetic in the regions with stronger currents. Consistent with previous studies [34-36], the magnitude of eddy kinetic energy (EKE) in WBCs is strongly dependent on model resolutions. In general, all of the simulations clearly delineate that high levels of EKE (> 500 cm2 s-2) concentrated in the vicinity of the major current systems, such as Kuroshio current, the Gulf Stream, the Brazil-Malvinas Confluence (BMC), Agulhas Current, the Antarctic Circumpolar Current (ACC), and the equatorial current system. However, compared to the result of Ding et al. (2022) [36] using daily data, the standard deviation of surface EKE at all resolutions seems quantitatively underestimated, partly owing to the smoothing of part of EKE by using monthly mean model data. Nevertheless, our results robustly indicate a substantial increase of surface EKE when the horizontal resolution increases from 15 km to 3 km, and the magnitudes of increase are not linear. For instance, compared to the EKE STD value of 153.1 cm2 s-2 at 15 km resolution (**Fig. S9a**), the surface EKE simulated at 10 km resolution increases by only 2%, with the EKE STD value as 156.5 cm2 s-2 (**Fig. S9b**), whereas it increases to a much larger extent, by 15% and 24% at grid spacings of 5 km (**Fig. S9c**) and 3 km (**Fig. S9d**), respectively. The submesoscale activities are clearly observed in the evolutions of eddies during intrusion of the Kuroshio into the South China sea (SCS) in the 5 km or higher resolution models (**Fig. S10**). In the 3 km resolution model, the daily-based snapshots and animation for the process of an eddy peeling off from the Kuroshio intrusion current clearly show activities of accompanying submesoscale vortex filaments (**Fig. S11** and **Animation S1**). Since the simulations at different resolutions are forced by identical atmospheric conditions, the enhancement in EKE along with resolution increase could be resulted from a stronger oceanic mean flow [37-39] with increased generation of meanders and eddies through flow instabilities [40, 41]. This suggests that increasing the spatial resolution of eddy-resolving models not only better simulates the intensity and position of ocean mean flow but also significantly enhances the energy of mesoscale and finer-scale eddies, which is worth to be further studied in future to make clear the behaviors of fine-scale interactions at higher-resolution models and their impacts on global weather-climate phenomena.

Text S6: More analyses on impacts of permitted submesoscales on SST simulations

As the capability of models to simulate submesoscale activities increases with increasing resolutions, the change of SST errors has strong geographical dependence (**Fig. S12**). While the SST errors consistently become smaller with higher model resolutions in the tropics and subtropics (40ºS-40ºN), the SSTs appear large uncertainties at high-latitudes. It is distinguishable that the SST errors in the high-latitude Southern Ocean and North Atlantic become larger as the model resolution is higher, but the errors in the region from the Norwegian

Sea to Barents Sea reduce as the model resolution is higher. The regions where with higher model resolutions SST errors become small or show large uncertainties correspond to small or large interannual SST variations in the spinup period (**Fig. S13**). To understand the role of eddy activities for local SSTs, a high-pass filter using a moving gaussian window with a cutoff wavelength of 50 km is applied to all data, and the SST errors are separated as mesoscales ($\leqslant$50 km) and largescales (> 50 km) by the 50 km horizontal spatial scale. In tropical and subtropical Oceans as well as the region from the Norwegian Sea to Barents Sea, the SST errors are dominant by the mesoscale part (**Fig. S14**) and the mesoscale and largescale parts have a consistent behavior as the model resolution increases (**Fig. S15**). Due to the dominant mechanism of eddy-mean flow interactions for local upper oceans in tropical and subtropical Oceans [42, 43] as well as the region from the Norwegian Sea to Barents Sea [44, 45], in a relatively-short (interannual) model spinup, well-represented fine-scale eddies in the high-resolution models improve the SST simulation [42, 46]. In these regions, due to complex topography features and the existence of WBCs, submesoscale eddies and fronts are usually very active [45, 47]. Nevertheless, for the Southern Ocean and North Atlantic, the interactions between the upper and deep Oceans [48] as well as the global thermohaline transport [49] play large roles, and therefore the largescale misfittings due to insufficient model spinup dominate the SST errors (see **Fig. S15**).

As an outstanding example of rich fine-scale eddies and eddy-mean flow interaction impacting SSTs, in regions of the Kuroshio and Kuroshio Extension and the Gulf Stream [50], the SST simulation errors become remarkably smaller as the model resolution increases, especially high up to permitting submesoscale activities (**Figs. S16-17**). It's worth to mention that although the relatively-short (3-year spinup) model integrations may not be appropriate for a systematical evaluation of SST bias as previous modeling studies [5], from error analyses above, we gain some understanding on the impacts of fine-scale eddy activities on SST simulation.

Text S7: Current model computational efficiency and ongoing optimization

The column 2 of **Table S3** lists the current computational efficiency of the series of developed coupled models based on 600 nodes (2400 core-groups). With aids of our optimization experiences on the Sunway TaihuLight [51], we first implement optimization of the version of 25v10 on the new Sunway system. Due to changes of the new Sunway on hardware and the codes of CESM2 from CESM1.3, most of the hotspots that were optimized on TaihuLight CPEs are not deliverable for the CESM2 on the new Sunway. We set two optimization phases for the newly-developed series of coupled models as shown in Columns 3 & 4 of **Table S3**. Optimization Phase I includes expanding allocation size and CPE parallel optimization of hotspots. Optimization

Phase II resolves the optimization issue of the fundamental data transfer function (malloc). Once the ultimate optimization target is done, these coupled ESMs with different high-resolutions can meet various needs with different computational costs.

References S1:

1. Fu H, Liao J and Yang J et al. The sun-way TaihuLight supercomputer: system and applications. Sci China Inform Sci 2016b; **59**: 072001.
2. Dongarra J. Sunway taihulight supercomputer makes its appearance. Natl Sci Rev 2016; **3**: 265-6.
3. Danabasoglu G, Lamarque JF and Bacmeister J et al. The Community Earth System Model Version 2 (CESM2). J Adv Model Earth Sy 2020; **12**: e2019MS001916.
4. Zhang S, Fu H and Wu L et al. Optimizing high-resolution Community Earth System Model on a heterogeneous many-core supercomputing platform (CESM-HR_sw1.0). Geosci Model Dev 2020; **13**: 4809-29.
5. Chang P, Zhang S and Danabasoglu G et al. An unprecedented set of high-resolution earth system simulations for understanding multiscale interactions in climate variability and change. J Adv Model Earth Sy 2020; **12**: e2020MS002298.
6. Golaz JC, Larson VE and Cotton WR. A PDF-based model for boundary layer clouds. Part I: Method and model description. J Atmos Sci 2002; **59**: 3540-51.
7. Larson VE. CLUBB-SILHS: A parameterization of subgrid variability in the atmosphere. 2017. arXiv:1711.03675v2 [physics.ao-ph].
8. Gettelman A and Morrison H. Advanced two-moment bulk microphysics for global models. part I: Off-line tests and comparison with other schemes. J Clim 2015; **28**: 1268-87.
9. Beljaars ACM, Brown AR and Wood N. A new parameterization of turbulent orographic form drag. Q J R Meteorol Soc 2004; **130**: 1327-47.
10. Sun Q, Whitney M M and Bryan FO et al. Assessing the skill of the improved treatment of riverine freshwater in theCommunity Earth System Model (CESM) relative to a new salinity climatology. J Adv Model Earth Sy 2019; **11**: 1189-206.
11. Li Q, Webb A and Fox-Kemper B et al. Langmuir mixing effects on global climate: WAVEWATCH III in CESM. Ocean Modelling 2016; **103**: 145-60.
12. Hunke EC, Lipscomb WH, Turner AK et al. CICE: The Los Alamos Sea Ice Model. Documentation and Software User's Manual. Version 5.1.T-3 Fluid Dynamics Group, Los Alamos National Laboratory, Tech. Rep. LA-CC-06-012, 2015.
13. Lawrence DM, Fisher RA and Koven CD et al. The Community Land Model Version 5: Description of new features, benchmarking, and impact of forcing uncertainty. J Adv Model Earth Sy 2019; **11**: 4245-87.
14. Hurrell JW, Holland MM and Gent PR et al. The Community Earth System Model: A framework for collaborative research. B Am Meteorol Soc 2013; **94**: 1339-60.

15. Lipscomb WH, Fyke JG and Vizcaíno M et al. Implementation and initial evaluation of the Glimmer Community Ice Sheet Model in the Community Earth System Model. J Clim 2013; **26**: 7352-71.
16. Fisher, RA, Wieder WR, and Sanderson BM et al. Parametric Controls on Vegetation Responses to Biogeochemical Forcing in the CLM5, J Adv Model Earth Sy 2019; **11**: 2879-95.
17. Ghimire B, Riley WJ, and Koven CD et al. Representing leaf and root physiological traits in CLM improves global carbon and nitrogen cycling predictions, J Adv Model Earth Sy 2016; **8**: 598-613.
18. Ali AA, Xu C, and Rogers A et al. A global scale mechanistic model of photosynthetic capacity (LUNA V1.0). Geosci Model Dev 2016; **9**: 587-606.
19. Brzostek ER, Fisher JB, and Phillips RP. Modeling the carbon cost of plant nitrogen acquisition: Mycorrhizal trade-offs and multipath resistance uptake improve predictions of retranslocation. J Geophys Res-Biogeo 2014; **119**: 1684-697.
20. Shi M, Fisher JB, and Brzostek ER et al. Carbon cost of plant nitrogen acquisition: Global carbon cycle impact from an improved plant nitrogen cycle in the Community Land Model. Global Change Biol 2016; **22:** 1299-314.
21. Wieder WR, Lawrence DM, and Fisher RA et al. Beyond Static Benchmarking: Using Experimental Manipulations to Evaluate Land Model Assumptions. Global Biogeochem Cy 2019; **33**: 1289-309.
22. Xu S, Ma J and Zhou L et al. Comparison of sea ice kinematics at different resolutions modeled with a grid hierarchy in the Community Earth System Model (version 1.2.1). Geosci Model Dev 2021; **14**: 603-28.
23. McClean JL, Bader DC and Bryan FO et al. A Prototype Two-Decade Fully-Coupled Fine-Resolution CCSM simulation. Ocean Model 2011; **39**: 10-30.
24. Murray R. Explicit Generation of Orthogonal Grids for Ocean Models. J Comput Phys 1996; **126**: 251-73.
25. Giraldo FX and Restelli M. A study of spectral element and discontinuous Galerkin methods for the Navier-Stokes equations in nonhydrostatic mesoscale atmospheric modeling: Equation sets and test cases. J Comput Phys 2008; **227**: 3849-77.
26. Dennis JM, Edwards J and Evans KJ et al. CAM-SE: A scalable spectral element dynamical core for the Community Atmosphere Model. International Journal of High Performance Computing 2012; **26**: 74-89.
27. Neale RB, Chen CC and Gettelman A et al. Description of the NCAR Community Atmosphere Model (CAM 5.0) (NCAR Technical Note NCAR/TN-486+STR). National Center of Atmospheric Research, 2012.
28. Lauritzen PH, Nair RD and Herrington AR et al. NCAR release of CAM-SE in CESM2.0: A reformulation of the spectral element dynamical core in dry-mass vertical coordinates with comprehensive treatment of condensates and energy. J Adv Model Earth Sy 2018; **10**: 1537-70.
29. Nair, RD, Levy MN and Lauritzen PH. Emerging Numerical Methods for Atmospheric Modeling. In: Lauritzen P, Jablonowski C, Taylor M and Nair R (eds). Numerical Techniques for Global Atmospheric Models. Lecture Notes in Computational Science and Engineering, vol 80. Springer, Berlin, Heidelberg,

2011.
30. Dagan G, Stier P and Dingley B et al. Examining the Regional Co-Variability of the Atmospheric Water and Energy Imbalances in Different Model Configurations-Linking Clouds and Circulation. J Adv Model Earth Sy 2021; **14**: e2021MS002951
31. Rios-Berrios1 R, Bryan GH and Medeiros B. Differences in tropical rainfall in aquaplanet simulations with resolved or parameterized deep convection. J Adv Model Earth Sy 2022; **14**: e2021MS002902.
32. Merlis TM and Held IM. Aquaplanet Simulations of Tropical Cyclones. Curr Clim Change Rep2019; **5**:185-95,
33. Zhang GJ and Mcfarlane NA. Sensitivity of climate simulations to the parameterization of cumulus convection in the Canadian climate centre general circulation model, Atmos ocean 1995; **33**: 407-46.
34. Bryan FO, Hecht MW and Smith RD. Resolution convergence and sensitivity studies with North Atlantic circulation models. Part I: The western boundary current system. Ocean Model 2007; **16**: 141-59.
35. Thoppil PG, Richman JG and Hogan PJ. Energetics of a global ocean circulation model compared to observations. Geophys Res Lett 2011; **38**: L15607.
36. Ding M, Liu H and Lin P et al. Overestimated Eddy Kinetic Energy in the Eddy-Rich Regions Simulated by Eddy-Resolving Global Ocean-Sea Ice Models. Geophys Res Lett 2022; **49**: e2022GL098370.
37. Smith RD, Maltrud ME and Bryan FO et al. Numerical simulation of the North Atlantic Ocean at 1/10. J Phys Oceanogr 2000; **30**: 1532-61.
38. Yu X, Zhang S and Li J et al. A Multi-Timescale EnOI-Like High-Efficiency Approximate Filter for Coupled Model Data Assimilation. J Adv Model Earth Sy 2019; **11**: 45-63.
39. Chassignet EP, Yeager SG and Fox-Kemper B et al. Impact of horizontal resolution on global ocean-sea-ice model simulations based on the experimental protocols of the Ocean Model Intercomparison Project phase 2 (OMIP-2). Geosci Model Dev 2020; **13**: 4595-637.
40. Thoppil PG, Richman JG and Hogan PJ. Energetics of a global ocean circulation model compared to observations. Geophys Res Lett 2011; **38**: L15607.
41. Oschlies A. Improved representation of upper-ocean dynamics and mixed layer depths in a model of the North Atlantic on switching from eddy-permitting to eddy-resolving grid resolution. J Phys Oceanogr 2002; **32**: 2277-98.
42. Waterman S, Hogg NG, and Jayne SR. Eddy-mean flow interaction in the Kuroshio Extension region. J Phys Oceanogr 2011; **41**: 1182-208.
43. Ma X, Jing Z, and Chang P et al. Western boundary currents regulated by interaction between ocean eddies and the atmosphere. Nature 2016; **53**5: 532-6
44. Vage S, Basedow SL, and Tande, KS et al. Physical Structure of Barents Sea Polar Front near Storbanken in August 2007. J Marine Syst 2014; **130**: 256-62.
45. Atadzhanova OA, Zimin AV, and Svergun, EI et al. 2018. Submesoscale Eddy Structures and Frontal Dynamics in the Barents Sea. Phys Oceanogr 2018; **25**: 220-28.
46. Yang H, Zhu R, and Chen Z et al. Temperature variability and eddy-flow interaction in the south of Oyashio Extension. J Geophys Res-Oceans 2022; **127**: e2022JC019051.

47. Bakhoday-Baskyabi M. Ocean surface hiden structures in the Lofoten area of the Norwegian Sea. Dynam Atmos Oceans 2020; **92**: 101173.

48. Macdonald AM, and Wunsch C. An estimate of global ocean circulation and heat fluxes. Nature 1996; **382**: 436-39.

49. Sakai K, and Peltier WR. A simple model of the Atlantic thermohaline circulation: Internal and forced variability with paleoclimatological implications. J Geophys Res 1995; **100**: 13455-79.

50. Ducet N. and Traon PYL. A comparison of surface eddy kinetic energy and Reynolds stresses in the Gulf Stream and the Kuroshio Current systems from merged TOPEX/Poseidon and ERS-1/2 altimetric data. J Geophys Res 2001; **106**: 603-16.

51. Gates WL, Boyle J and Covey C et al. An Overview of the Results of the Atmospheric Model Intercomparison Project (AMIP I). Bull Amer Meteor Soc 1998; **73**: 1962-70.

52. Kerry C, Rougham M. and Powell B. Predicting the submesoscale circulation inshore of the East Australian Current. J Marine Syst 2020; **204**: 103286.

53. Jing Z, Fox-Kemper B and Cao H et al. Submesoscale fronts and their dynamical processes associated with symmetric instability in the Northwest Pacific Subtropical Ocean. J Phys Oceanogr 2021; **51**: 83-100.

54. Bonanno E, Burgos D and Verstraete R et al. Inventorying USGS oceanographic geospatial datasets for inclusion at NOAA's national centers for environmental information. NOAA Technical Memorandum NOS CS 44, Earth Resources Technology and NOAA's Integrated Ocean and Coastal Mapping Program, Silver Spring, Maryland, USA, 2020.

55. Steele M, Morley R and W. Ermold, W PHC: A global ocean hydrography with a high quality Arctic Ocean J. Climate, 2001; 14: 2079-2087.

Figures S1-S20:

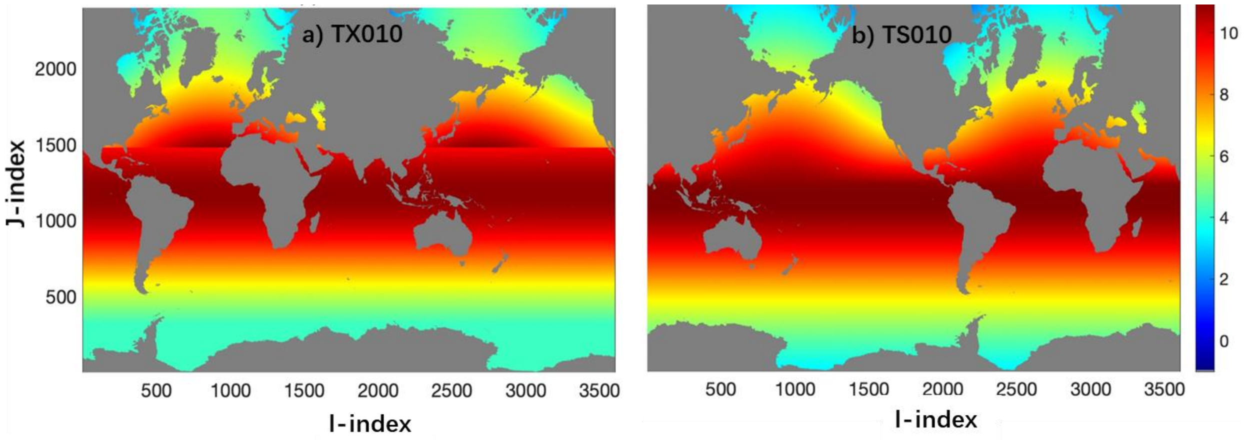

Figure S1. The distributions of the Height of T-cell East (HTE) in the *a*) traditional TX and *b*) new TS gridding systems with nominal 0.1° resolution.

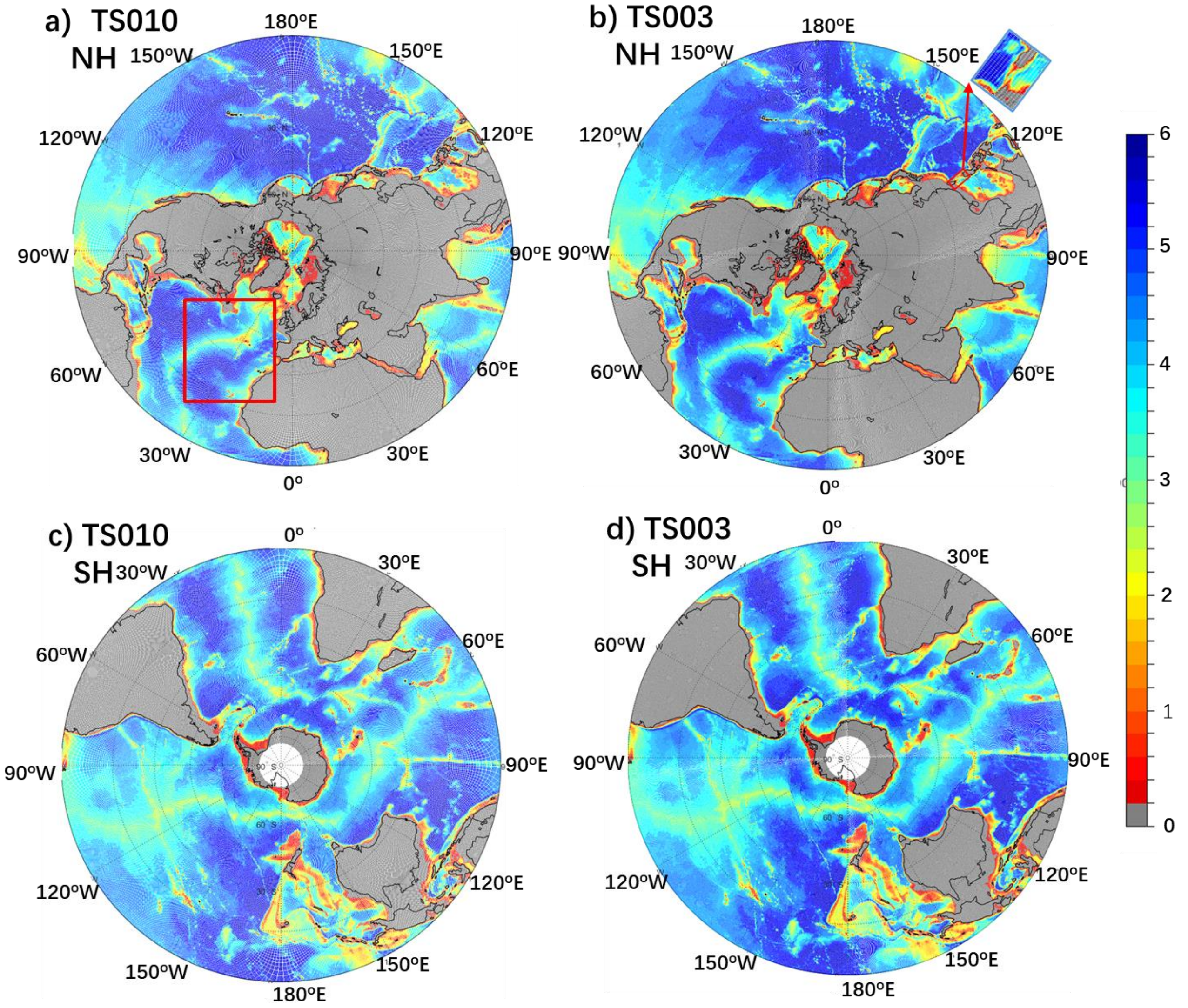

Figure S2. The distributions of submarine topography (unit: km) in *ab*) TS010, cd) TS003 models for Northern Hemisphere (NH, upper panels a and c), and Southern Hemisphere (SH, lower panels b and d). Red boxes denote the roomed-in regions for the Atlantic Middle Ridge and Luzon Strait shown in **Fig. S3**. The shown model submarine

topography is the result of applying smoothing technique [52, 53] to the 1 arc-minute ($\frac{1}{60}°$) resolution Earth Topography and Bathymetry dataset ETOPO1 [54].

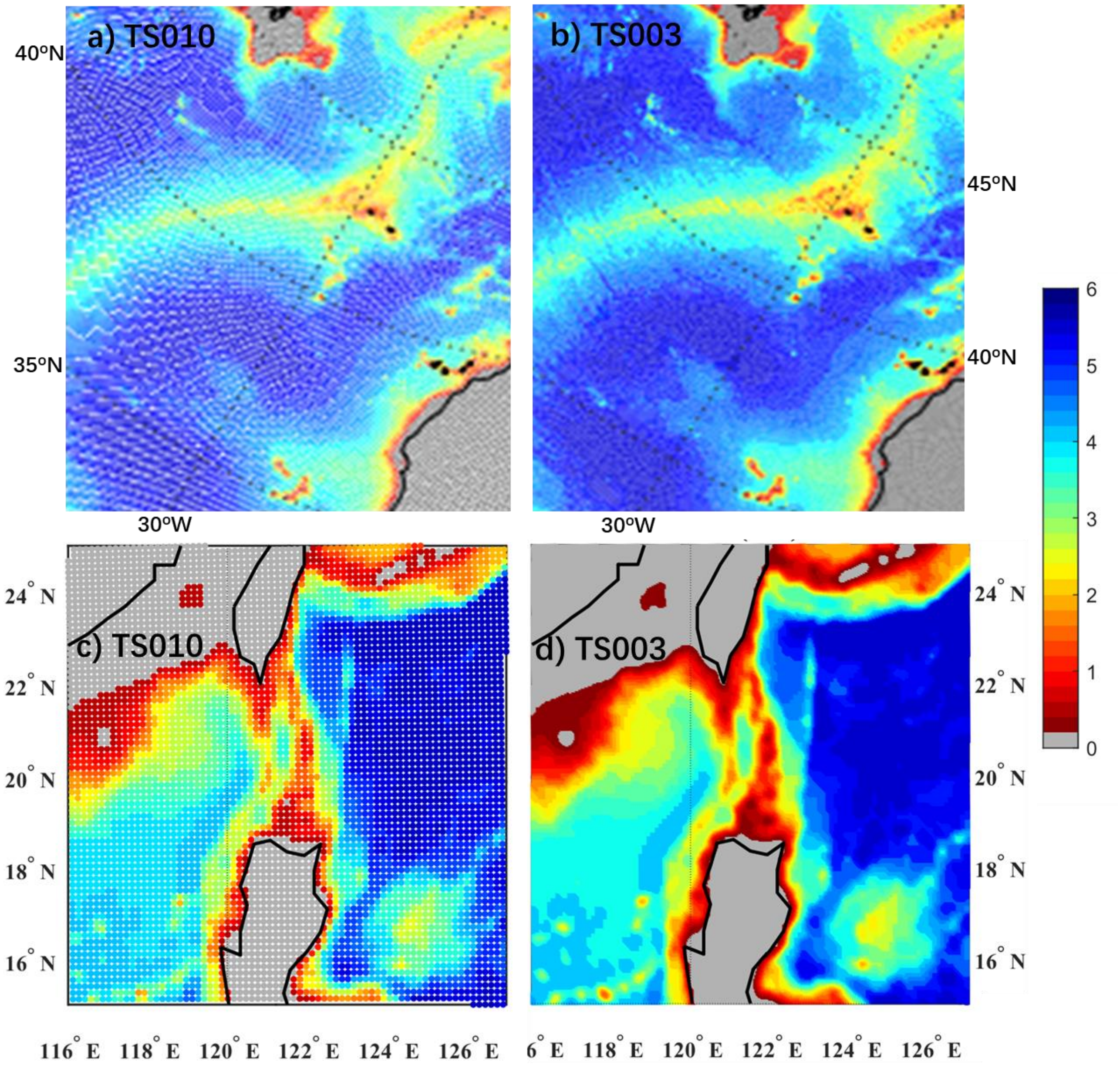

Figure S3. Same as **Fig. S2** but for *ab*) the North Atlantic Middle Ridge region and cd) Luzon Strait.

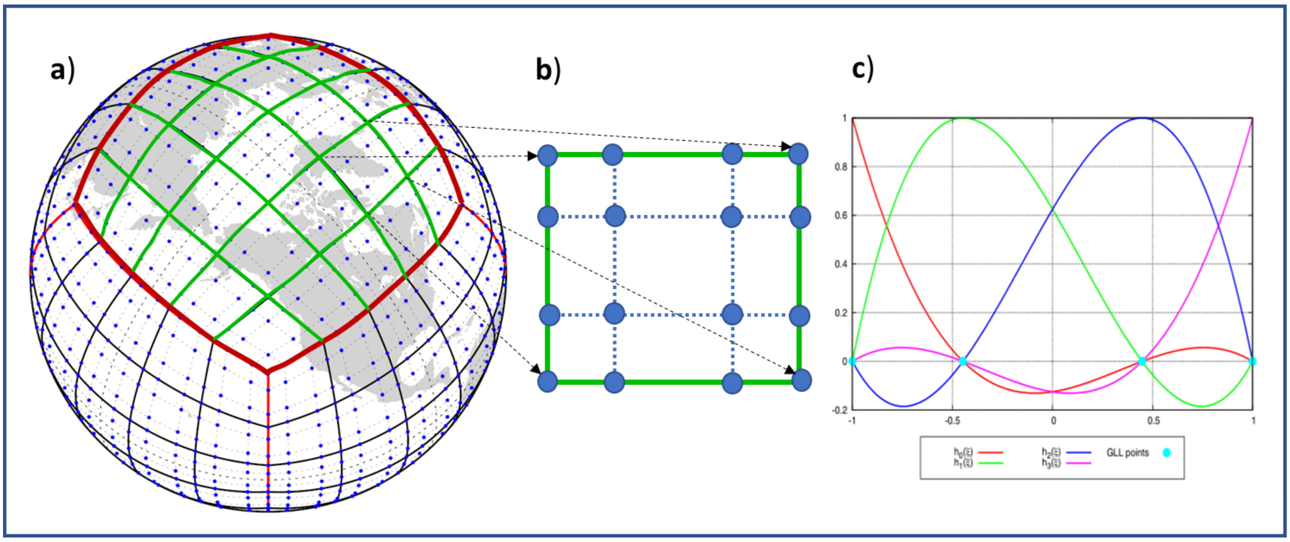

Figure S4. An illustration of Spectral Element (SE) method to discretize the atmosphere motion equations on a cubic sphere mesh system, as *a*) the cubic sphere grid structure for ne5np4 configuration, *b*) the grid-point (blue dots) and grid-box (squares and rectangles bounded by green segments) and *c*) Lagrange-Legendre polynomials of fixed degree k = N = 4 in the discontinuous Galerkin (DG) method [29] as the basis functions $h_{0,1,2,3}(\xi)$ of spectral expansion, whose zeros are at the Gauss-Lobatto-Legendre (GLL) quadrature points (cyan dots). The nodal version of DG employs GLL quadrature points, which are in the interval as $\xi \in [-1,1]$.

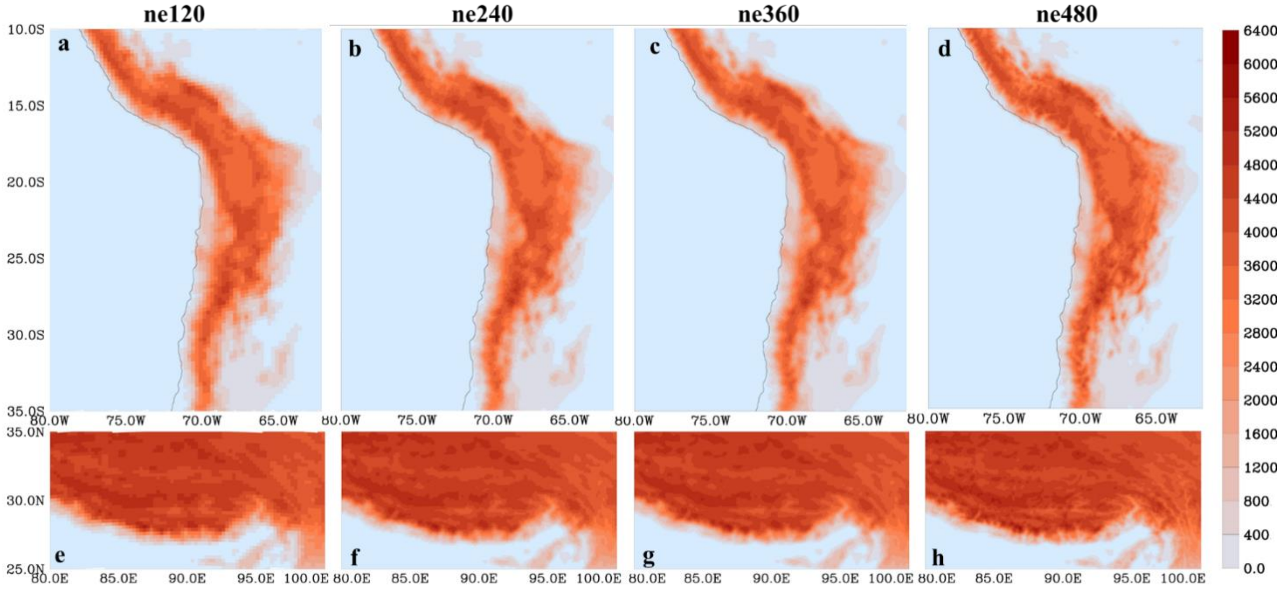

Figure S5. The geographical distributions of terrain geopotential heights (unit: gpm) of *abcd*) Andes and *efgh*) Himalaya mountains in ne120 (panels *a, e*) ne240 (panels *b, f*), ne360 (panels *c, g*) and ne480 (panels *d, h*) CAM-se models as the sub-samples of statistics shown in **Fig. S6**.

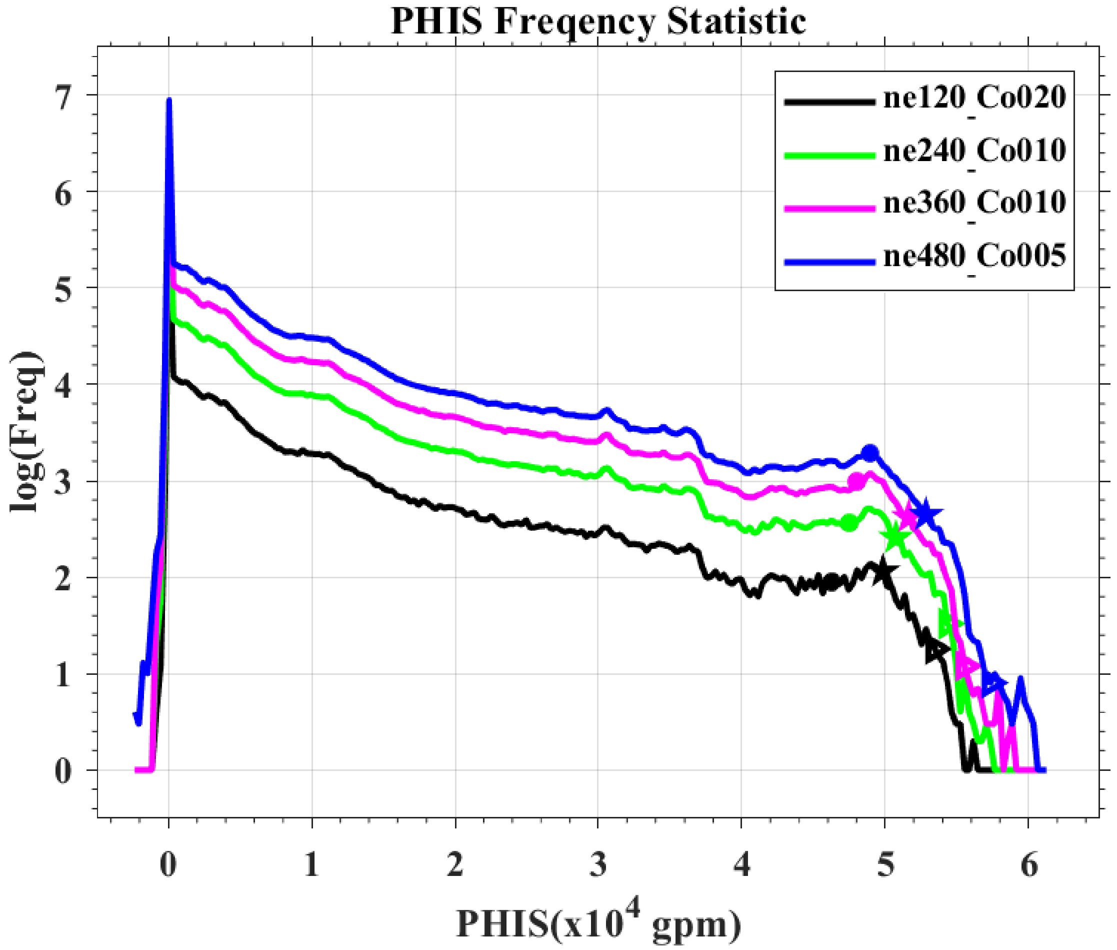

Figure S6. The variations of the logarithm of frequency (unit: grid-points) in ne240 (green), ne360 (purple) and ne480 (blue) CAM-se models in the space of terrain potential heights (unit: gpm), denoted with corresponding 90% (dot), 95% (asterisk) and 99% (triangle) percentile in each model. The curve of existed ne120 CAM-se model (black) is plotted as a reference. Note that different resolutions use a slightly different smoothing coefficient to smooth out sampling topography spikes.

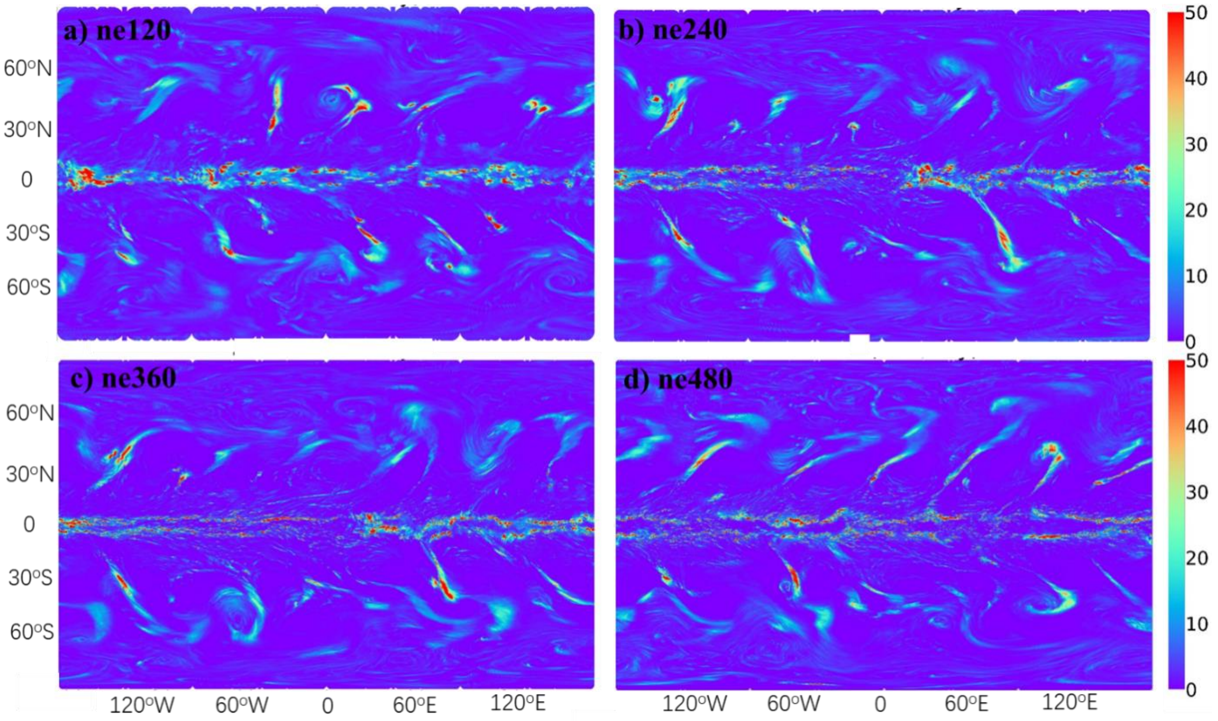

Figure S7. The distributions of the convective precipitation rates (unit: mm/day) produced by the community atmosphere model with the *a*) ne120, *b*) ne240, *c*) ne360 and *d*) ne480 spectral element dynamical core (CAM-SE) in an aqua-planet experiment framework.

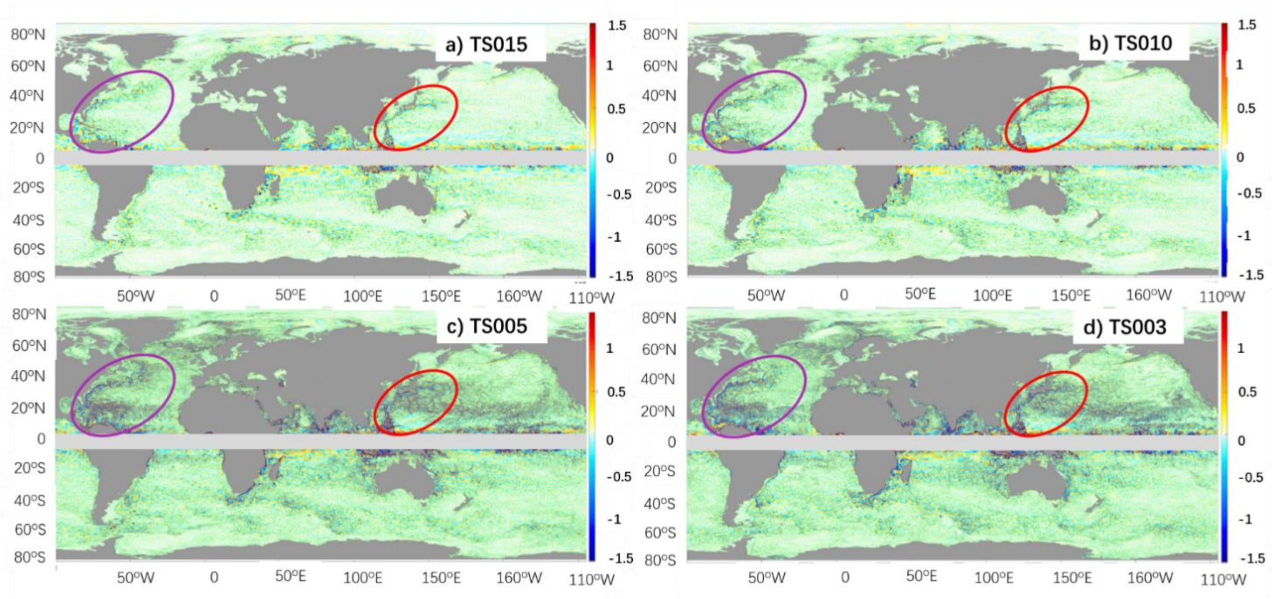

Figure S8. The distributions of sea surface vorticity at four different resolutions ocean and sea-ice coupled models of nominal *a*) 0.15°, *b*) 0.1°, *c*) 0.05° and *d*) 0.03°, after 3-year spinup starting from the Polar Science Center Hydrographic Climatology (PHC) ([55] Steele et al., 2001) and forced by climatological surface fluxes. What is shown excludes the equatorial region where the Coriolis parameter *f* is close to zero.

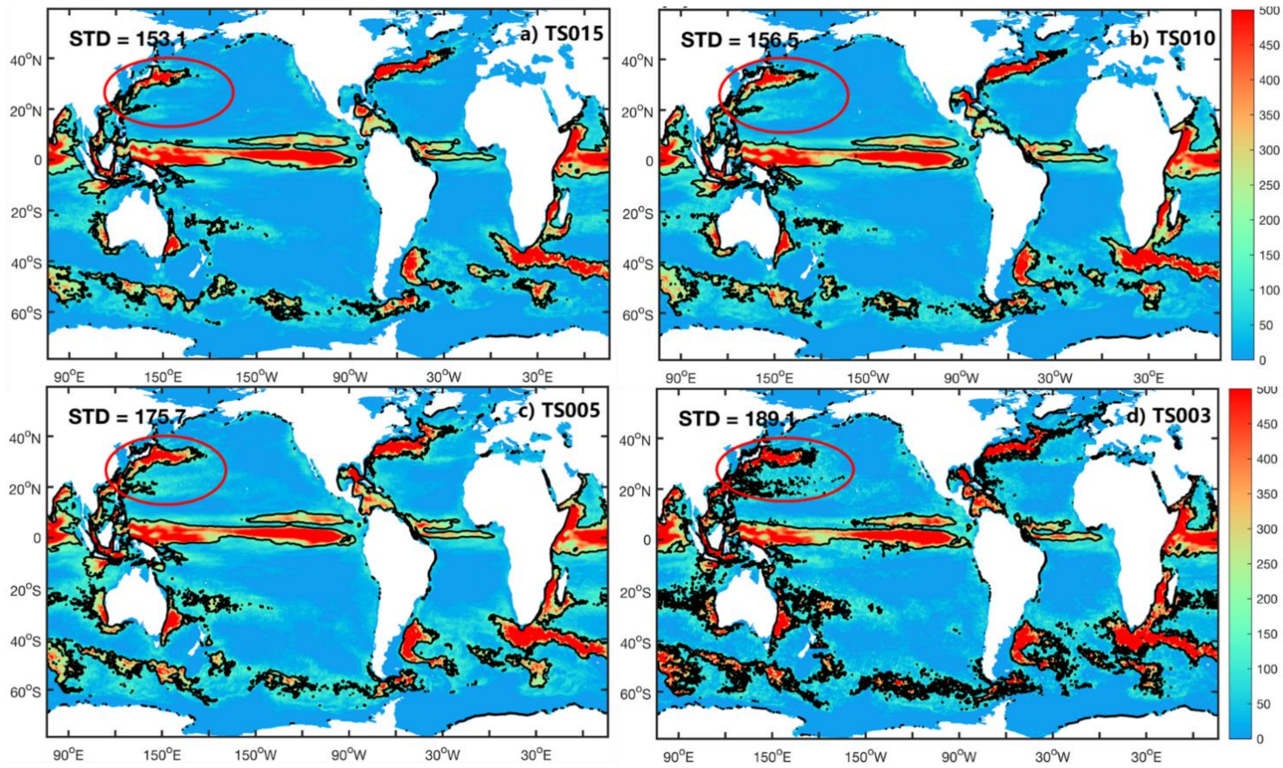

Figure S9. The distributions of eddy kinetic energy in *a*) TS015, *b*) TS010, *c*) TS005 and *d*) TS003 sea-ice coupled models at four different resolutions as 0.15°, 0.1°, 0.05° and 0.03°., computed by monthly data in the last two years of 3-year spinup model integrations.

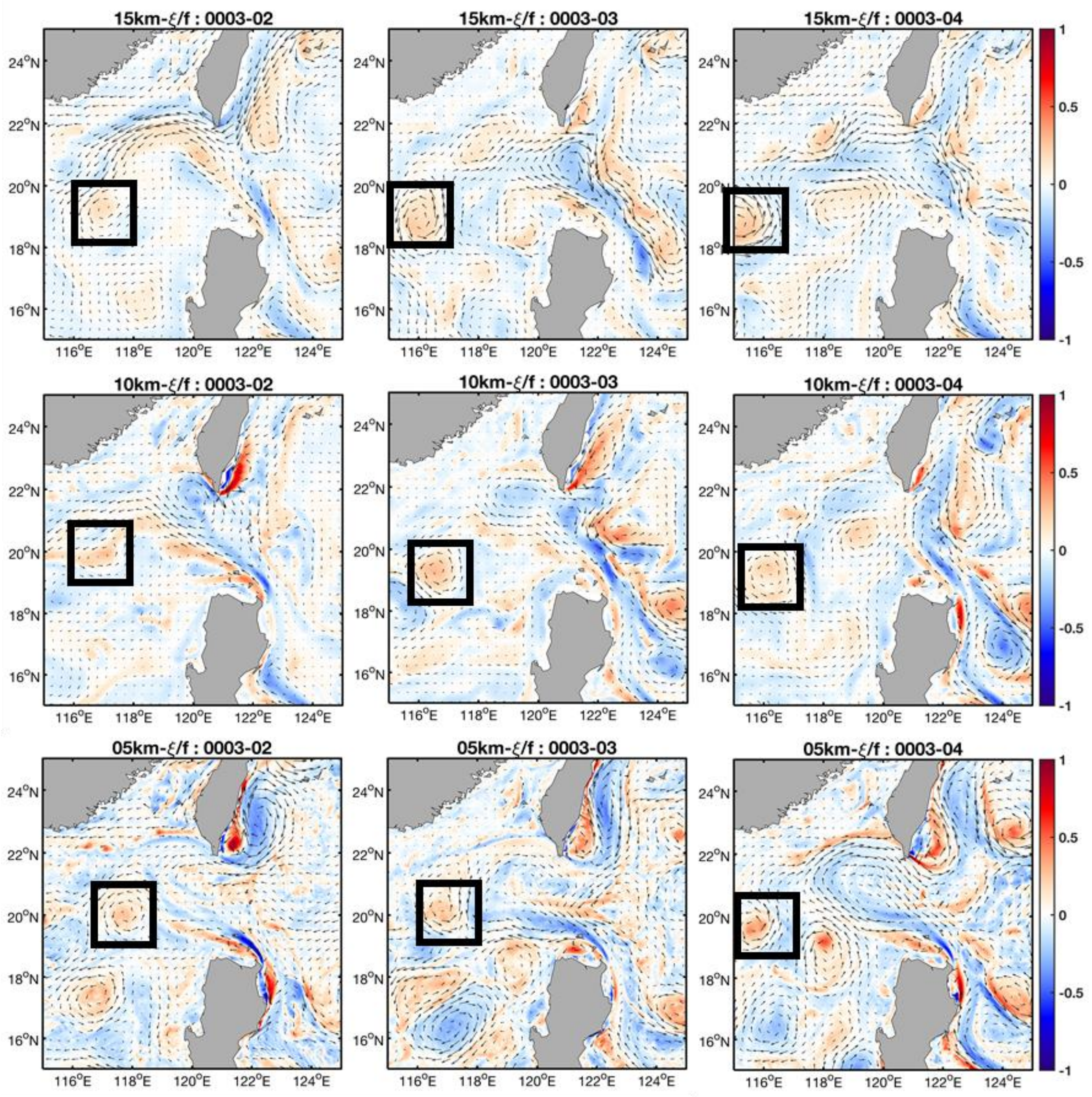

Figure S10. Monthly evolutions of a cyclonic eddy (black box) process during intrusion of the Kuroshio into the South China sea (SCS) in the TS015 (upper), TS010 (middle) and TS003 (lower) models during their spinup integrations. The color-shade and arrow respectively show surface normalized relative vorticity and geostrophic velocity anomalies.

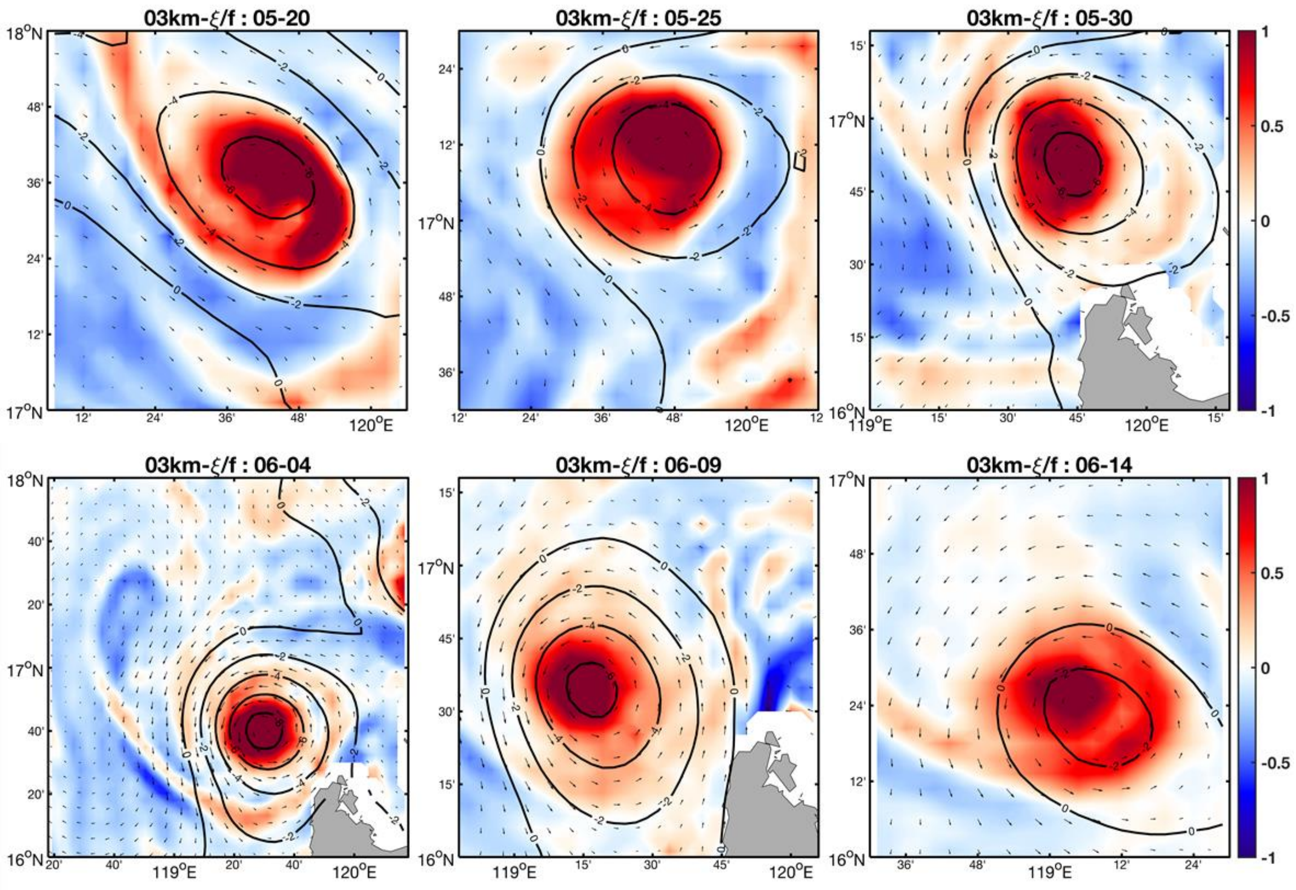

Figure S11. 6 snapshots in a CE peeling off process from the Kuroshio intrusion current of the TS003 model simulation, drawn from **Animation S1**. The color-shade, contours and arrows respectively show surface normalized relative vorticity, SLA (unit: cm), and geostrophic velocity anomalies.

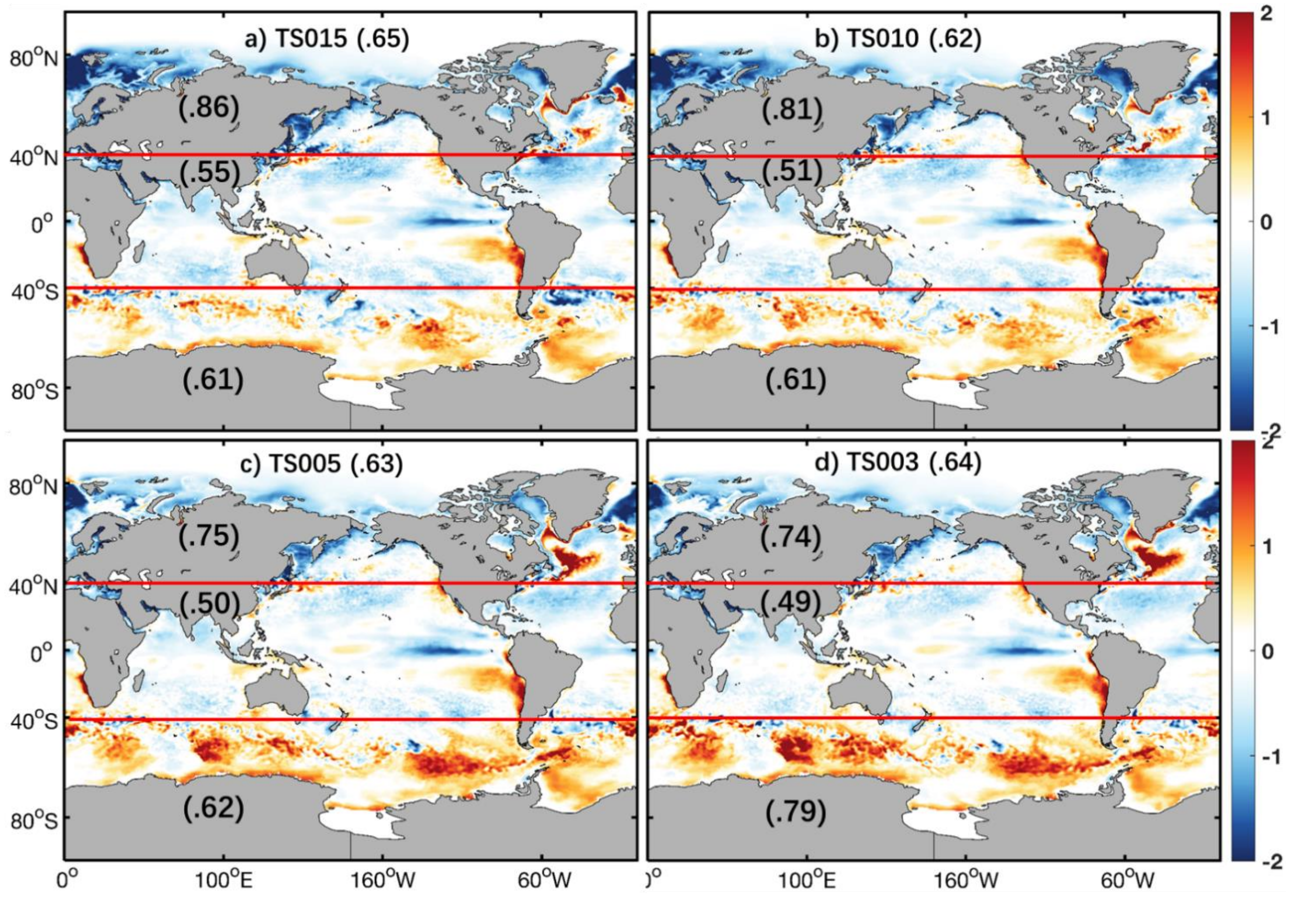

Figure S12. The distributions of SST mean errors (unit: °C) in model simulations by *a*) TS015, b) TS010, c) TS005 and d) TS003 in the last year of 3-year spinup, against the 0.25° resolution satellite observational product AVHRR (the Advanced Very High Resolution Radiometer) (https://www.eumetsat.int/avhrr). The numbers in parentheses in each panel are the root mean square (rms) in the corresponding latitudes separated by the red segments.

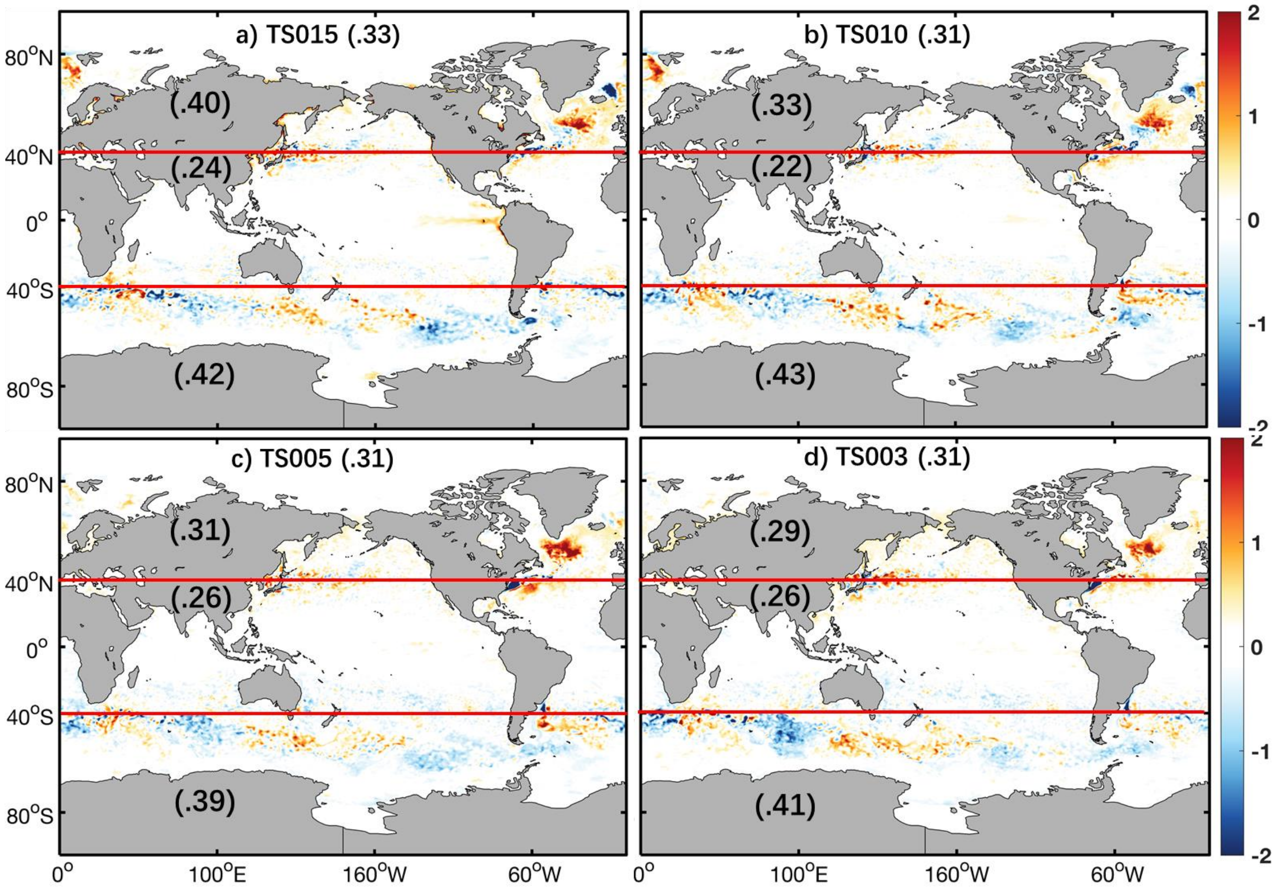

424

425 **Figure S13.** The same as **Fig. S12** but for the SST differences (unit: °C) between the 3rd-year and 2nd-year model simulations. The numbers in parentheses in each panel are the root mean square (rms) in the corresponding latitudes separated by the red segments.

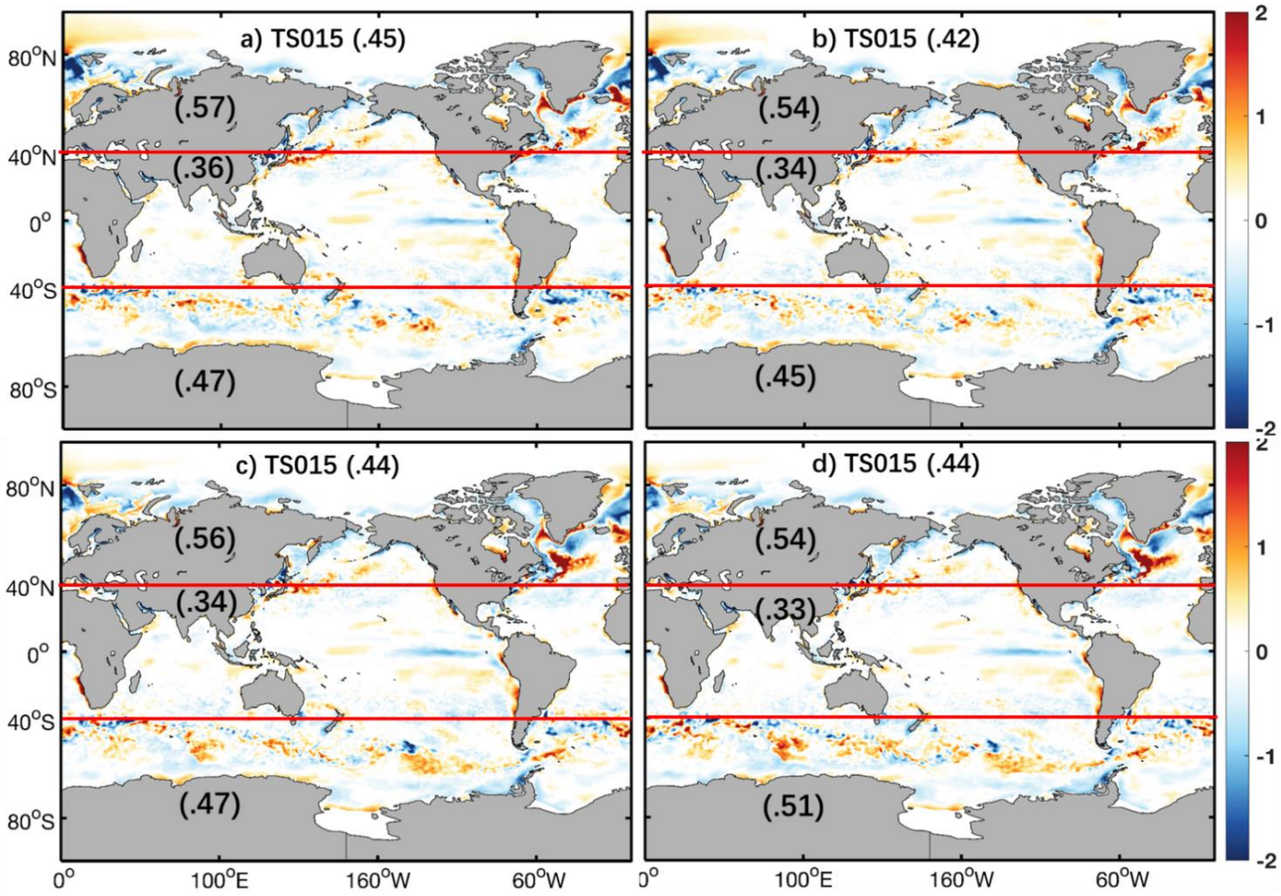

Figure S14. The same as **Fig. S12** but for mesoscale signals. All data are spatially high-pass filtered using a moving gaussian window with a cutoff wavelength of 50 km. After spatially filtering, the maps in this figure only contain features with wavelengths ⩽ 50 km considered as the mesoscale signals.

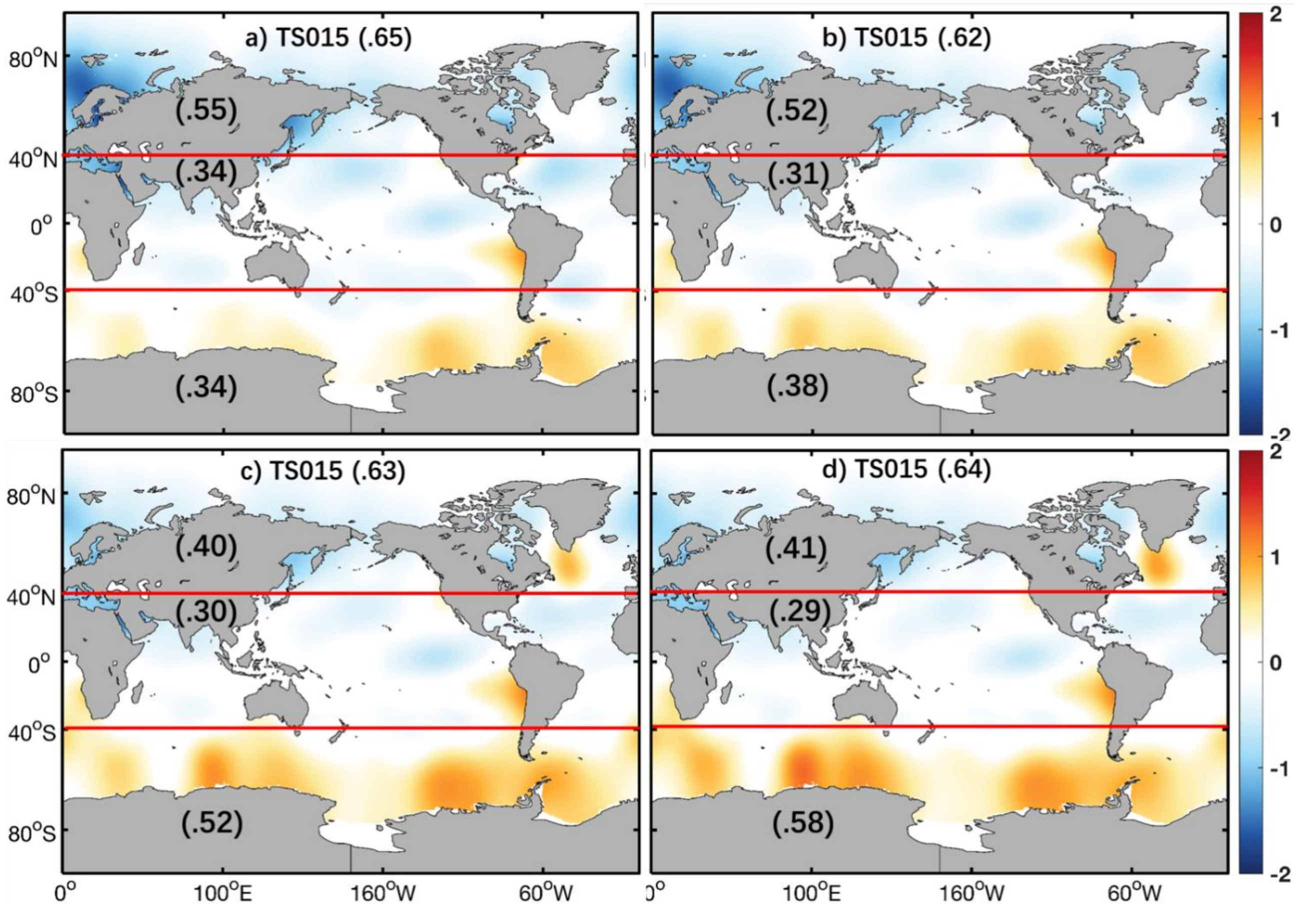

Figure S15. The same as **Fig. S14** but for largescale signals that are the differences between the full and mesoscale fields and of SST errors (i.e. features with wavelengths > 50 km).

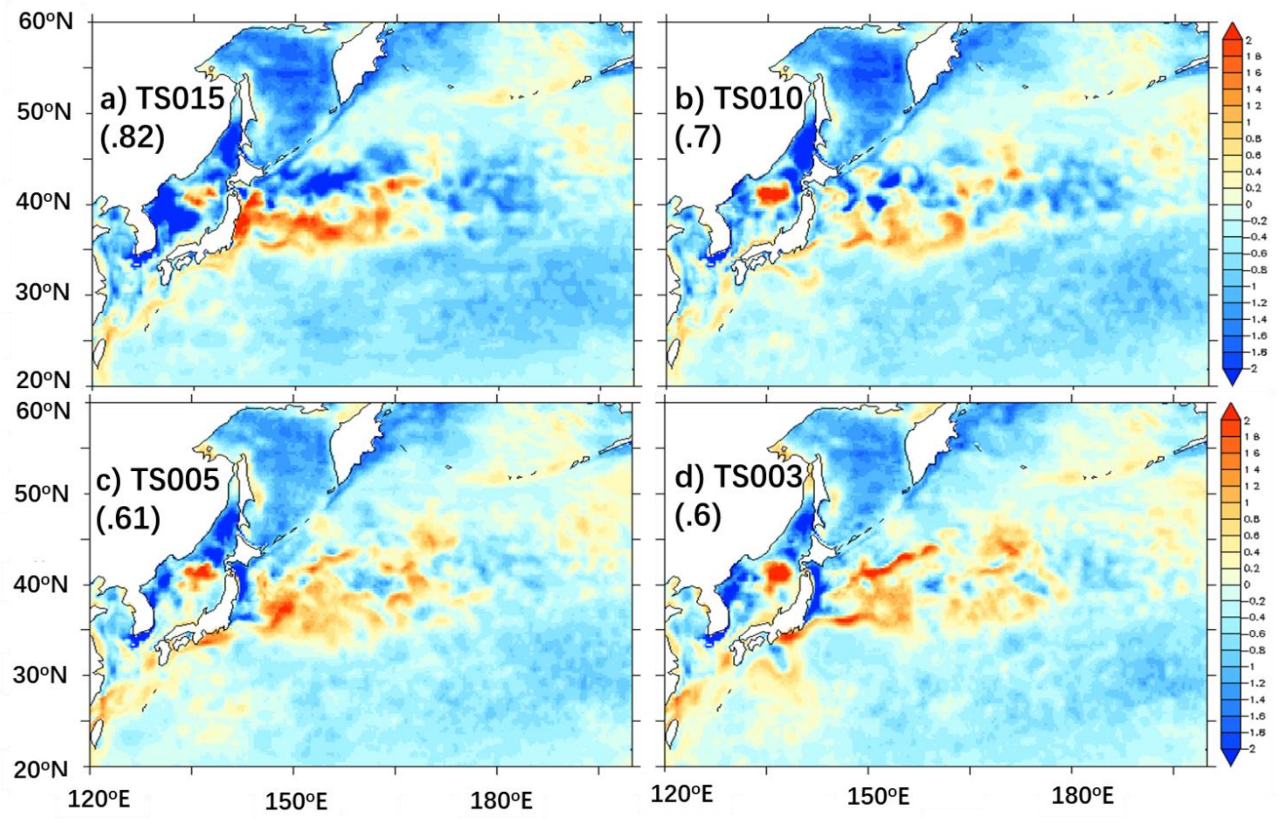

Figure S16. The distributions of SST mean errors (unit: °C) in the Northwest Pacific of model simulations by *a*) TS015, b) TS010, c) TS005 and d) TS003 in the last year of 3-year spinup. The number in the parenthesis in each panel is the corresponding root mean square (rms).

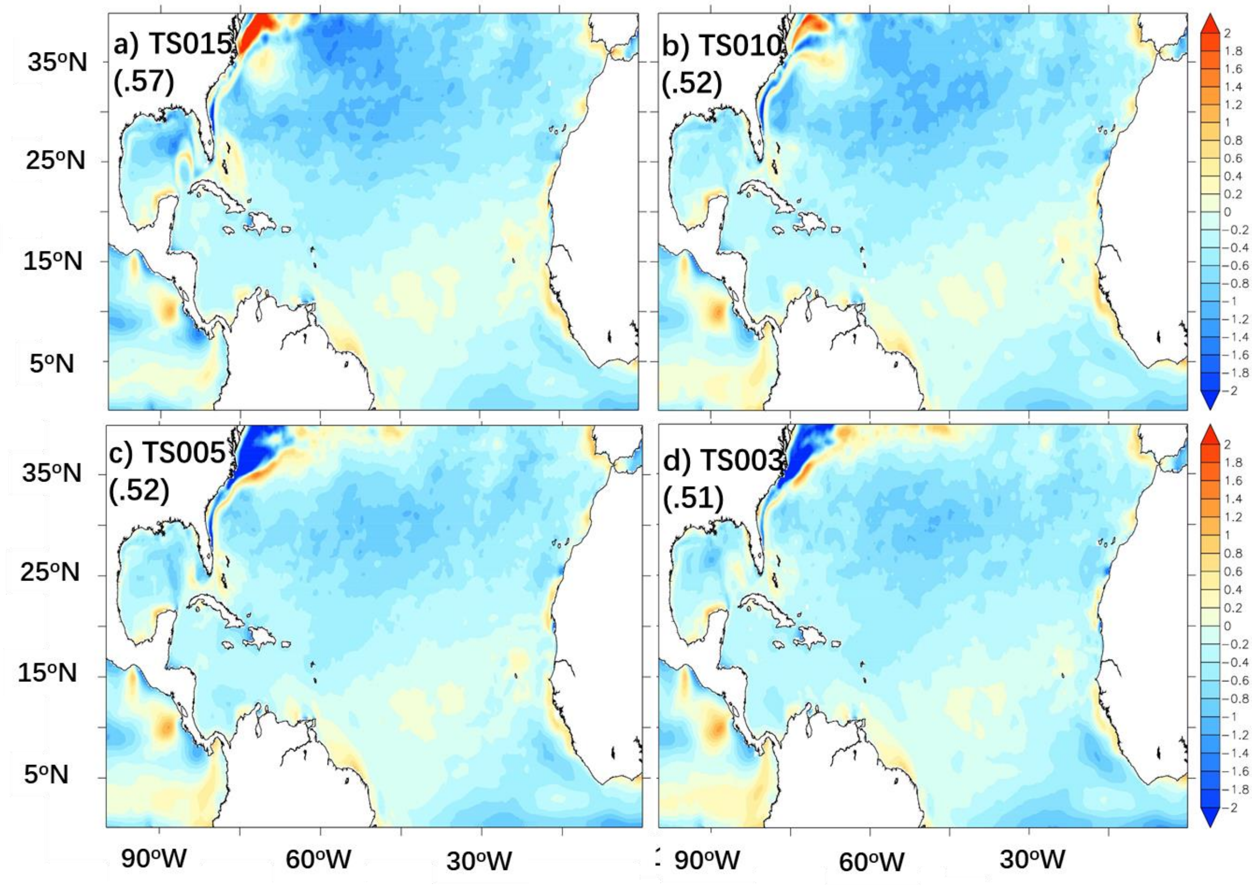

Figure S17. The same as **Fig. S16** but for tropical Atlantic.

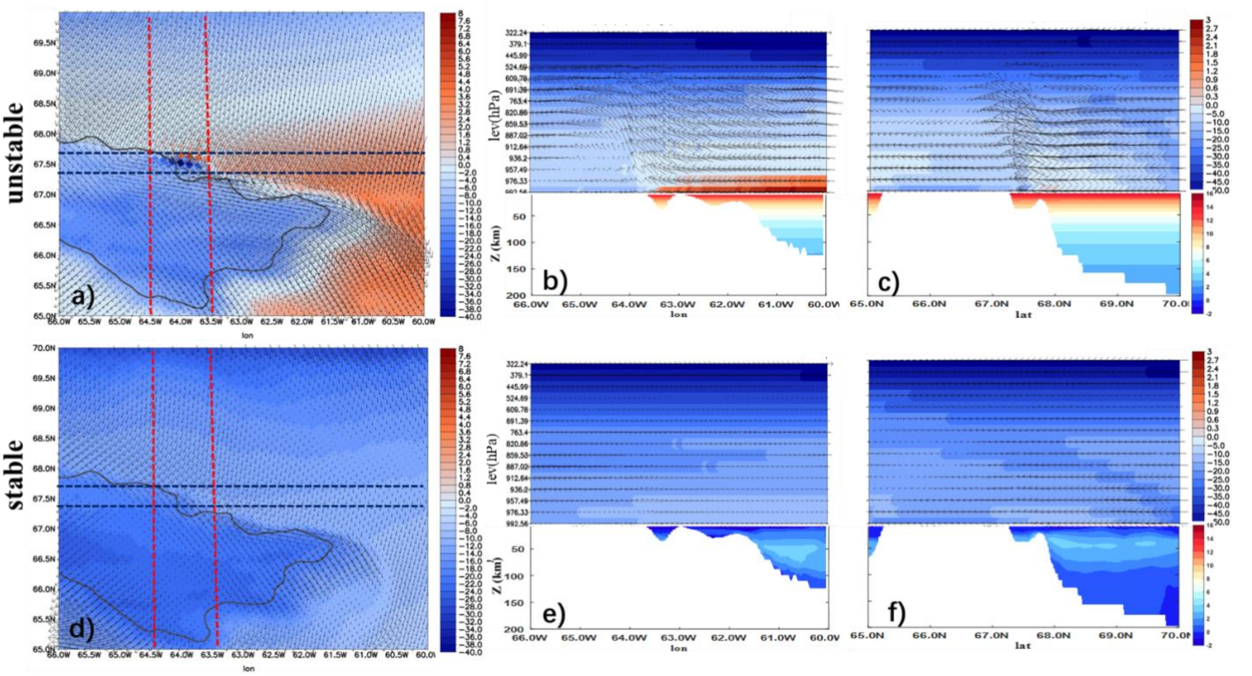

Figure S18. An example of the a) computational instability developed in the high-resolution coupled model 5v3 (ne480 CAM-SE coupled with the 0.03° resolution POP ocean model) due to *bc*) the shocks at the air-sea interface excited by

different spinup time scales of the atmosphere and ocean. The instability originates from the coastal line in the intersection area of double dotted-black lines and dotted-red lines in panel *a* and develops in the south coastal area of the Davis Strait appeared as abnormally strong convections induced by abnormally high sea-surface temperatures as shown in the zonal (panel *b*) and meridional (panel *c*) channels bounded by the dotted-black lines and dotted-red lines in panel *a*. The corresponding counterparts of stable situation after the ocean model is spun-up for a few months are plotted in panels *d*-*f*. In all panels, the color-shaded is temperature (unit: °C) and the arrow represents the [u,v] (panels a and d) or [v,$10^{-2}\times$ w]/[u,$10^{-2}\times$w] (panels b and c)/(panels e and f) vector of the atmosphere flows (unit: m/s).

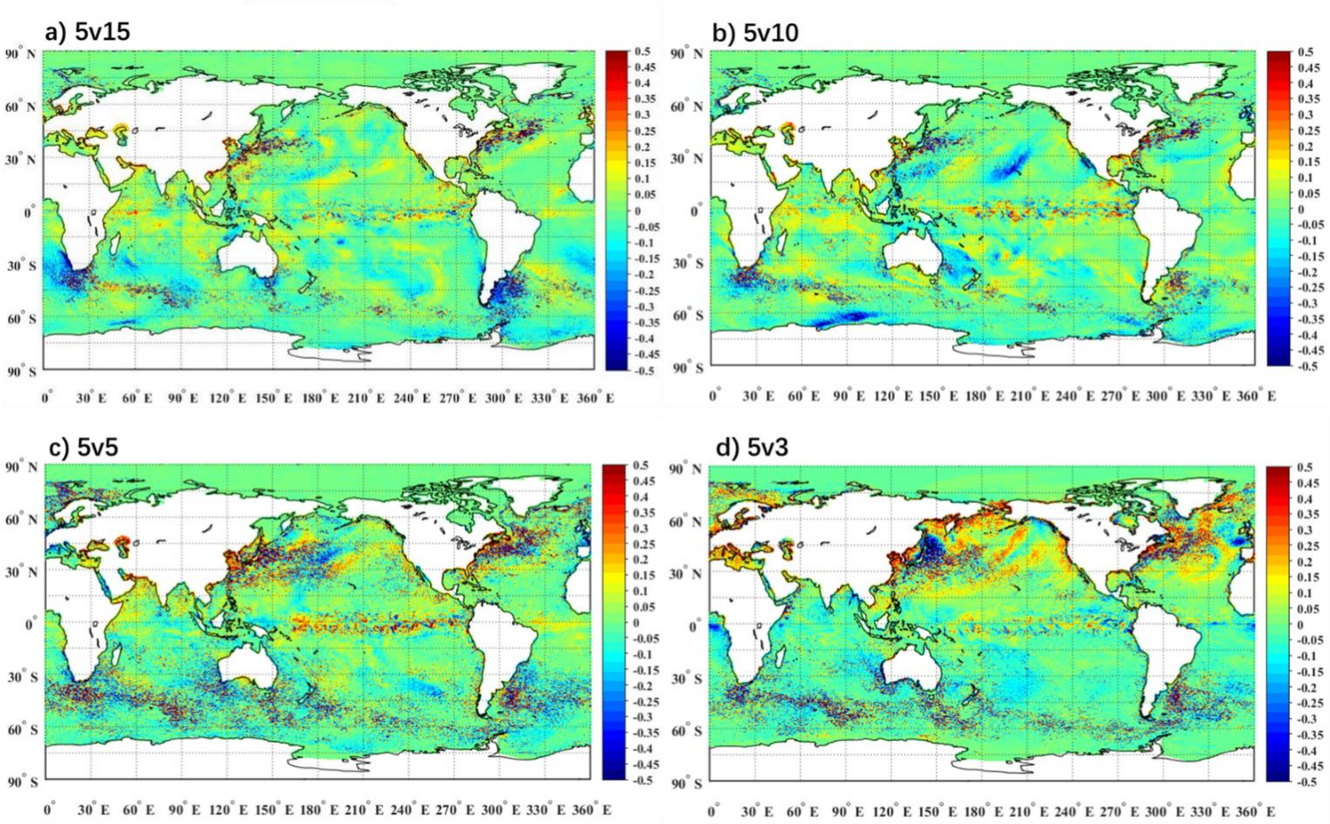

Figure S19. The distributions of SSTs produced by *a)* 5v15, *b)* 5v10, *c)* 5v5 and *d)* 5v3 models in the last month of the half year of spinup integrations.

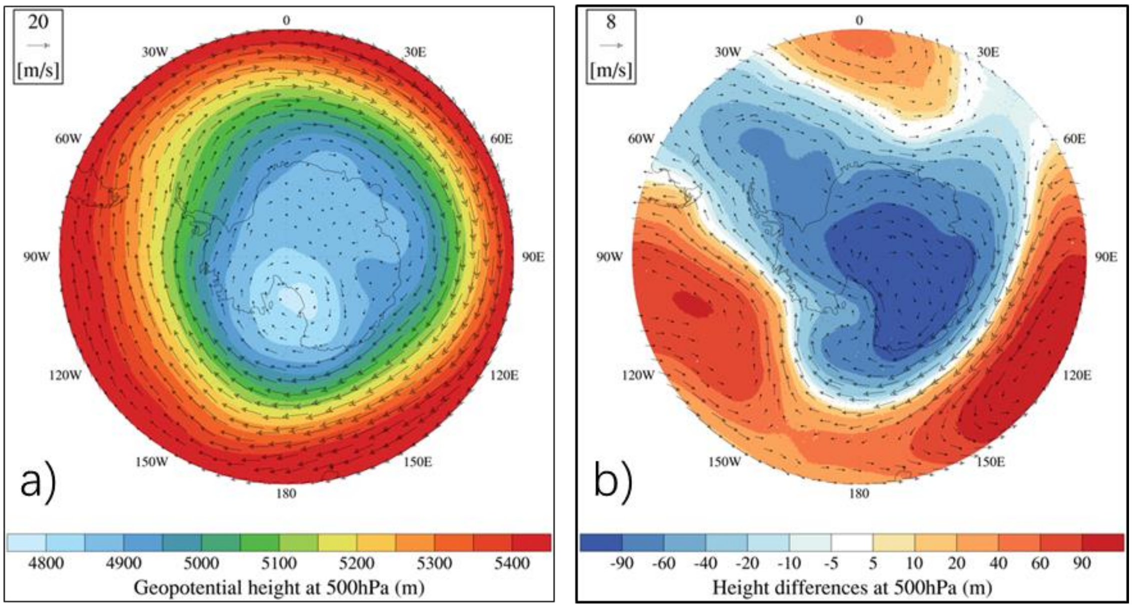

Figure S20: Spatial distributions of a) 500 hPa geopotential heights (GHTs) and winds from the iAMAS with global uniform 3-km (U3km) resolution simulation and b) the difference of 500 hPa GHTs and winds between U3km and U60km iMAMS simulations over the Southern hemisphere

Tables S1-S3:

Table S1. List of grid-spacing properties of POP models with different resolutions in the new tripolar TS gridding configurations.

POP Configuration	Total gridpoints	Gridpoints at the equator	Grid-scale (km) at the equator	Grid-size (km) at the equator	Mean grid-scale (km) at 65°N
TS015	2400×1200	2400	23.58	16.68	9.78
TS010	3600×1800	3600	15.73	11.12	6.50
TS005	7200×3600	7200	7.86	5.56	3.27
TS003	12000×6000	12000	4.72	3.34	1.96

Table S2. List of grid-spacing properties of CAM-SE models in different configurations of cubic sphere elements*.

Name in CAM-SE	Total gridpoints	Total gridboxes	Gridpoints at the equator	Mean cell length (km) along the equator	Global mean (min, max) cell size (km)
ne120np4	777602	777600	1440	27.8	25.6 (22, 30)
**ne240np4(pg3)**	**3110402**	**3110400**	**2880**	**14.0**	**12.8 (11, 15)**
**ne360np4(pg3)**	**6998402**	**6998400**	**4320**	**9.27**	**8.54 (7.4, 9.8)**
**ne480np4(pg3)**	**12441602**	**12441600**	**5760**	**6.95**	**6.4 (5.6, 7.4)**

*Note: The ne120np4 are the existed model as information reference and the bold-black ones are new development in this study.

Table S3. Current computational efficiency and ongoing optimization on heterogeneous many-core HPCs*.

Model Name	Current Computational Cost (2400 Core-Groups) (MPEs-only)	Optimizing Phase I Target (72000 Core-Groups) (MPEs+CPEs)	Optimizing Phase II Target (72000 Core-Groups) (MPEs+CPEs)
25v10	~25	~5×365	~8×365
12v5	~8	~1.5×365	~2.4×365
9v5	~5	~1×365	~1.6×365
5v15	~3.5	~.75×365	~1.2×365
5v10	~3	~.6×365	~1×365
5v5	~2.5	~.5×365	~.8×365
5v3	~2	~.3×365	~.5×365

*Note: Here, for comparison, the unit to measure computational efficiency is simulation days per day (SDPD). Optimizing Phase I includes expanding allocation size optimization and CPE parallel optimization, and Optimizing Phase II adds the optimization of a fundamental data transfer function (malloc).

Animation S1:

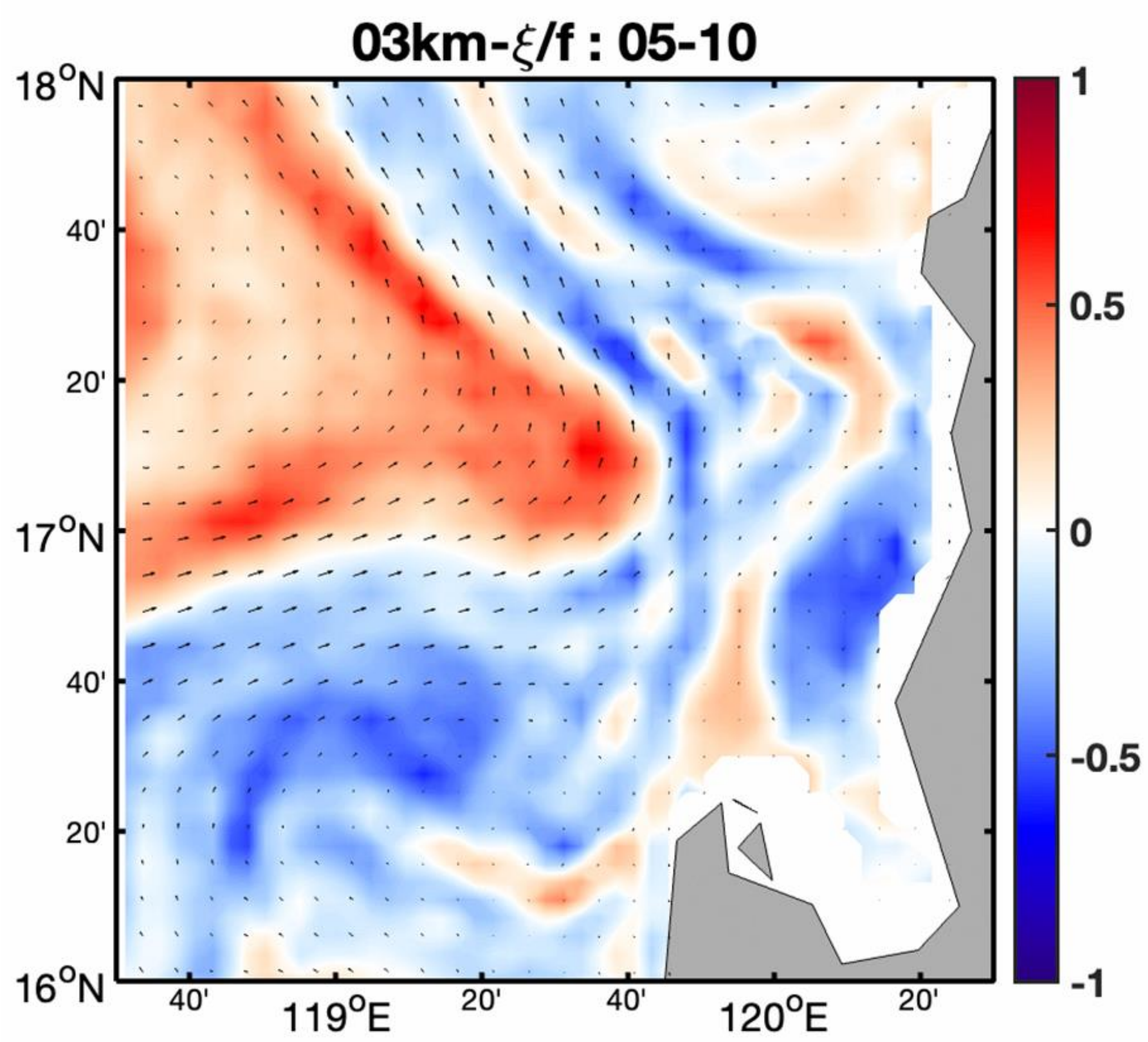

Animation S1. An animation for a CE peeling off process from the Kuroshio intrusion current in TS003 model during 10 May to 25 June, which accompanies with submesoscale activities simulated by the submesoscale eddy permitting model. The color-shade and arrows respectively show surface normalized relative vorticity and geostrophic velocity anomalies.
