# Supplementary figures and images for: Toward Earth system modeling with resolved clouds and ocean submesoscales on heterogeneous many-core HPCs
Source: Natl Sci Rev. 2023 Mar 20;10(6):nwad069. doi: 10.1093/nsr/nwad069 (PMC10171631; doi:10.1093/nsr/nwad069)

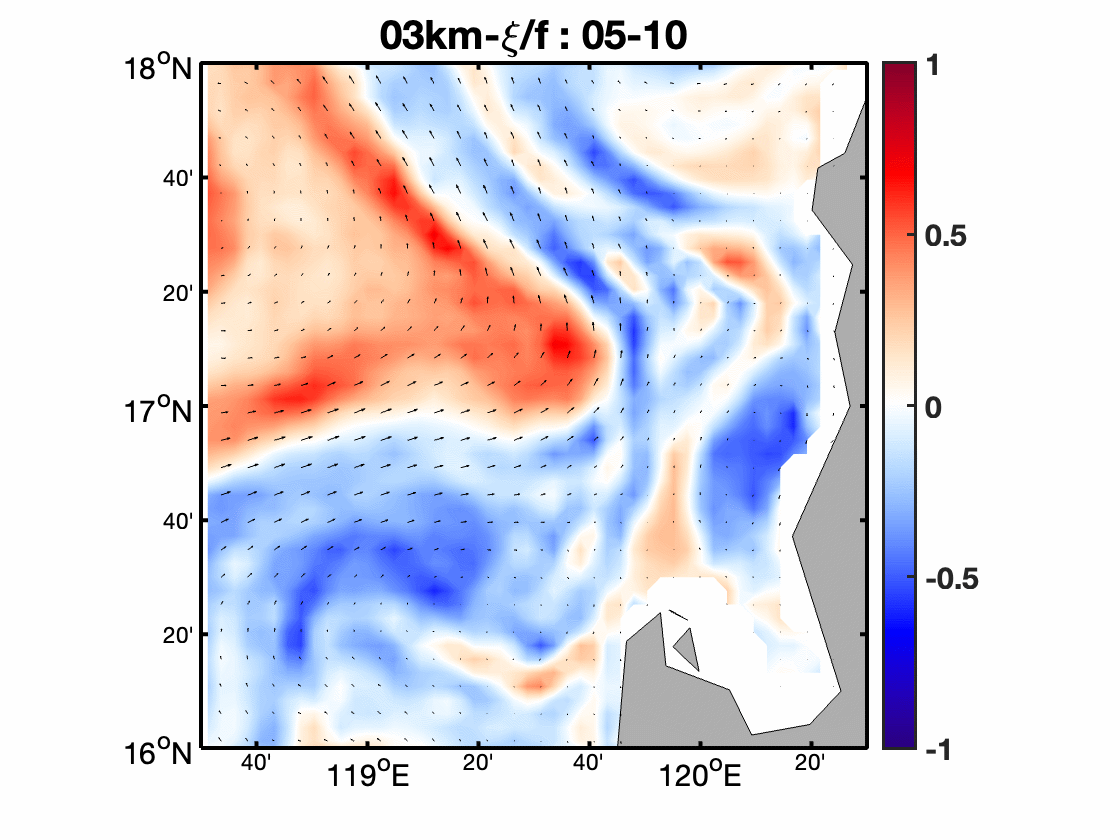

Supplement: nwad069_Supplemental_Files [file nwad069_supplemental_files.zip › NSR_MS-2022-1185.Supplementary_Animation_S1.gif]
